# Preterm Birth Conditions Alter Muscle Stem Cells and Their Niche, Causing Lasting Impairments in Muscle Regeneration and Function

**DOI:** 10.1002/jcsm.70058

**Published:** 2025-09-16

**Authors:** Alyson Deprez, Thomas Molina, Gael Cagnone, Pauline Garcia, Séverine Leclerc, Anik Cloutier, Rebecca Desaulniers, Benjamin Ellezam, Anne Monique Nuyt, Nicolas A. Dumont

**Affiliations:** ^1^ CHU Sainte‐Justine Azrieli Research Center Montreal Canada; ^2^ Faculty of Medicine, Department of Pharmacology and Physiology Université de Montréal Montreal Canada; ^3^ Faculty of Medicine, Department of Pediatrics Université de Montréal Montreal Canada; ^4^ Faculty of Medicine, School of Rehabilitation Université de Montréal Montreal Canada

**Keywords:** inflammation, muscle stem cells, myogenesis, preterm birth, regeneration

## Abstract

**Background:**

Preterm birth‐related conditions affect the development of multiple organs, such as the heart, the lungs and the brain, leading to long‐term alterations in their function and a higher risk of comorbidities. Emerging evidence also indicates that the skeletal muscles are affected. We aimed to understand the mechanisms underlying these changes in skeletal muscles.

**Methods:**

A rodent model of transient neonatal hyperoxia and muscle samples of human babies born at term or preterm were used to investigate the impact of preterm birth‐related conditions on muscle stem cells, the engine of muscle growth and repair. Single cell transcriptomics, in vitro culture of myoblasts or single myofibres, ex vivo muscle contractile properties and in vivo experiments (cardiotoxin‐induced muscle injury) were performed to determine the impact of preterm birth on muscle stem cell function and regenerative capacity.

**Results:**

Preterm birth‐related conditions reduced the muscle stem cell pool from the newborn stage (−30%, *p* = 0.0134) until adulthood (−56%, *p* < 0.0001), along with impaired myogenic capacity and regenerative potential. In vitro analysis from rats showed impaired self‐renewal and reduced myotube size (−28.8%, *p =* 0.004). Human samples suggest a similar trend towards smaller myotube size in muscle stem cells from infants born at the earlier gestational age. Single‐cell RNA‐seq on rat samples revealed an enriched TNF‐α/NF‐κB signalling pathway within subsets of muscle stem cells. This pathway, mediated in part by interaction with macrophages, influences muscle stem cell fate decisions and myogenic trajectories. Culture experiments showed that myotubes treated with conditioned medium from macrophages of rats exposed to hyperoxia have reduced diameters (−66.5%, *p =* 0.0216). Early administration of an inhibitor of TNF‐α (Infliximab) restored the muscle stem cell pool postinjury (63%, *p* = 0.0073) and regenerative capacity.

**Conclusions:**

Overall, preterm birth‐related conditions promote an inflammatory microenvironment that disrupts the muscle stem cell pool and their function. This mechanism could explain the muscle atrophy and weakness observed in individuals born preterm and suggests potential therapeutic strategies to improve overall health outcomes in this population.

## 1 Introduction

Skeletal muscle, which represents 35–40% of an adult's body mass, is an important determinant of physical function and quality of life [1]. Skeletal muscle mass and function can be affected by many conditions such as cancer cachexia or age‐related sarcopenia, leading to impaired physical function, reduced quality of life and a higher mortality rate [1].

Each year, about one in 10 infants worldwide is born prematurely, defined as birth before 37 weeks of gestation (WG). Individuals born preterm are at increased risk of chronic conditions, including diabetes [2], cardiovascular diseases and neurodevelopmental impairments [3, 4]. This vulnerability is exacerbated with the degree of prematurity: moderate (32–36 WG), very preterm (28–32 WG) or extremely preterm (< 28 WG) [5, 6].

Accumulating evidence also suggests that skeletal muscles are affected by preterm birth‐related conditions [7]. During the late second to third trimester, the foetal skeletal muscle undergoes a critical phase of maturation [8, 9]. Premature birth abruptly exposes the muscle to the relatively hypoxic in utero environment to 21% O_2_ in ambient air, or even higher levels when ventilatory support is required, thereby constituting a hyperoxic insult that can induce long‐lasting detrimental effects on muscle development. Previously, using a well‐recognized animal model mimicking preterm birth‐related conditions, we reported that transient neonatal exposure to high oxygen levels resulted in a muscle phenotype resembling premature ageing such as oxidative stress, low‐grade inflammation, reduced fibre size, increased deposition of collagen and decreased muscle strength [10]. In humans, our findings from a cohort of adults born preterm versus at term showed similar alterations in skeletal muscle composition (reduced muscle area and increased stiffness) and function (reduced handgrip and knee extension strength) in preterm born individuals [11]. A systematic review and meta‐analysis support these findings, indicating muscle wasting/weakness in individual born preterm [7]. However, the underlying mechanisms explaining this phenotype are still elusive.

Postnatal muscle growth and muscle regeneration rely mainly on muscle stem cells (MuSCs) [12]. After a mechanical stress or growth stimulus, MuSCs emerge from their state of quiescence to become proliferating myoblasts, which later withdraw from the cell cycle to self‐renew or differentiate and fuse to form muscle fibres [13]. MuSCs are highly sensitive to the variation of their microenvironment, which could influence their function and cell fate decision. For instance, the chronic inflammatory state associated with ageing impairs MuSC expansion and regenerative capacity [14].

In this study, we used single cell RNAseq, in vitro cell culture (human and rat myogenic cells) and in vivo rodent model (rat exposed to hyperoxia in neonatal period) to study the impact of preterm birth on MuSCs. We report that preterm birth‐related conditions induce a low‐grade inflammatory niche marked by elevated expression of the potent proinflammatory cytokine TNF‐*α* and activation of the downstream NF‐κB pathway, a central transcriptional regulator of inflammation. This deregulation impairs MuSC myogenic progression and alters cell fate decisions, leading to long‐lasting deficits in muscle regeneration. The MuSC pool and regenerative capacity can be restored by treatment with infliximab, an inhibitor of TNF‐*α*. Altogether, these findings uncover a new mechanism by which MuSC defects contribute to the muscle phenotype observed in adults born preterm and identify a new therapeutic avenue to improve health in the preterm born population.

## 2 Methods

### 2.1 Neonatal Human Biopsy

Neonatal human biopsies were obtained from three infants born preterm (one female at 26.3 WG and two males at 32 and 33.4 WG) and one infant born at term (male at 37.4 WG). The female newborn born preterm died 9 days after birth (necrotizing enterocolitis), the male born preterm at 33.4 WG died 1 day after birth (diaphragmatic hernia), the male born at 32 WG died 5 days after birth (sepsis *E. coli* ) and the infant born at term died 2 days after birth (congenital cardiopathy). The inclusion criteria are to be born preterm (< 37 WG) or at term (> 37 WG) and died in the CHU Sainte‐Justine after minimum of 24 h of birth for preterm. Exclusion criteria are suspected (under investigation) or diagnosed congenital muscular pathology. A piece of the vastus lateralis biopsy was taken by a pathologist specialized in neuromuscular disorders as quickly as possible after death (15 h for the preterm male born at 32 WG, 18 h for the male born at 33.4 WG, 109 h for the female born at 26.3 WG and 15 h for the male born at term). Previous studies have shown that MuSCs can retain their regenerative capacities for many days postmortem (S1–S3). MuSCs were isolated from human muscle biopsies and cultured in vitro to evaluate their proliferation and differentiation capacities. All procedures have been approved by the institutional review board and have been performed in accordance with the ethical standards laid down in the 1964 Declaration of Helsinki and its later amendments (#2023‐4386). All parents of the babies gave their informed consent prior to their inclusion in the study.

### 2.2 Human Primary Myoblast Culture

Muscle biopsies from controls and preterm infants were minced and plated in a culture dish with foetal bovine serum (FBS) (S4). Myoblasts were grown and expanded in Sk‐MAX media complemented (Sk‐MAX supplement and 20% FBS). Myoblasts were purified by fluorescence‐activated cell sorting (FACS) using the CD56 AF647‐conjugated antibody (BD biosciences, clone R19‐760, 1:10) as described previously (S5). The differentiation of myoblasts was induced with 2% horse serum in DMEM low glucose media. Control and preterm myoblasts were used for experiments at the same low passage.

### 2.3 Animals

Sprague–Dawley (Charles River, Canada) pups were maintained with a dam in 80% O_2_ (OI, oxygen‐induced injury group) in an oxycycler (A82OCV, Biospherix) or in room air (CTRL, control group) from days 3–10 of life. To avoid maternal O_2_ toxicity, every 12 h, the dams in hyperoxia were interchanged with a dam from a litter maintained in room air. No more than four animals per litter (two males and two females, chosen randomly) were used for each experimental procedure. During the transient neonatal hyperoxia exposure, no mortality of pups or dams was observed, and no pups were excluded from the experimental procedure. All protocols were previously approved by the animal care committee of the Centre Hospitalier Universitaire Sainte‐Justine (#2023‐5044) and respected the principles of the Canadian Council on Animal Care Guide for the Care and Use of Experimental Animals.

### 2.4 Experimental Procedures

Animals were weighed prior to the sacrifice (decapitation under anaesthesia by inhalation of isoflurane, 2%–3%/L O_2_) at 10 days (neonatal), 4 weeks (juvenile) and 16 weeks (adult) postnatal, and the tibialis anterior (TA) was collected. For the TNF‐α inhibitor treatment, the rats received an i.p. injection of Infliximab (Y0002047 Sigma‐Aldrich) 5 mg/kg (S6) or vehicle (0.9% NaCl) at 10‐ and 20‐day‐old rats. For the muscle regeneration assessment, rats were anaesthetized by inhalation of isoflurane, 2%–3%/L O_2_ (and subcutaneous injection of buprenorphine at 0.3 mg/mL, 0.05 mg/kg of body weight) and the TA and the extensor digitorum longus (EDL) were injured by an intramuscular injection of cardiotoxin (10 μM, Latoxan) at 4 and 16 weeks of age.

### 2.5 Rat Primary Myoblast Culture

Major hindlimb muscles (e.g., TA, gastrocnemius and quadriceps) were collected from 4‐week‐old CTRL and OI rats and dissociated in collagenase/dispase solution using the gentleMACS dissociator (Miltenyi Biotech) as described previously (S7). After the digestion, the mononuclear cells were filtered through a 100 μm nylon strainer, and the red blood cells were removed (Biolegend, Lysis buffer, 420301). MuSCs were purified by FACS using the negative lineage selection in PE‐Cy7 for CD45, CD31 and TER‐119 (Biolegend) and positive lineage selection in FITC for integrin‐α7 (OriGene) (S8). Cells were cultivated in matrigel coated plate with DMEM (supplemented with glucose and pyruvate) + 1% Penicillin and streptomycin and 20% FBS. Myoblast differentiation was induced by exposure to low‐serum medium (DMEM + 5% horse serum + 1% penicillin and streptomycin).

### 2.6 Single Cell RNA Sequencing

Cells were isolated from major hindlimb muscles (left and right) as describe above from four males at 4 weeks of age (two CTRL and two OI). MuSC enrichment was performed by FACS using negative selection for CD45 (Biolegend, 202205), CD31 (Abcam, ab33858) and CD11B (Biolegend, 201805) FITC‐conjugated antibodies. The 115 000 cells obtained with 75%–80% of viability were immediately processed for single‐cell RNAseq using the 10X Genomics Chromium IX platform. ScRNAseq libraries were prepared using the 3′ CellPlex Reagent Kits v3.1 (10x Genomics; Pleasanton, CA, United States) according to the manufacturer's instructions. Generated libraries were sequenced on an Novaseq6000. Bioinformatics analyses are described in detail in the Supporting Information.

### 2.7 Additional Methods

Additional details regarding the methods related to myofibre isolation, immunostaining, western blot, ELISA and muscle function are provided in the Supporting Information.

### 2.8 Statistics

All experiments were repeated independently in the laboratory with similar results. For the different experiments, rats were randomly assigned to the different groups. Normality of the data was assessed using the Shapiro–Wilk test. Comparisons between groups were performed by two‐way ANOVA with Tukey's honest statistical difference for multiple comparison or two‐tailed Student's *t* test to compare quantitative data populations with normal distribution and equal variance with GraphPad Prism Version 7.0. Details of the tests used are described in the figure legends. The level of significance was set at *p* < 0.05. Results are expressed as mean ± SEM.

## 3 Results

### 3.1 Reduction of the MuSC Pool and Regenerative Capacity After Neonatal Exposure to Preterm Birth‐Related Conditions

**FIGURE 1:**
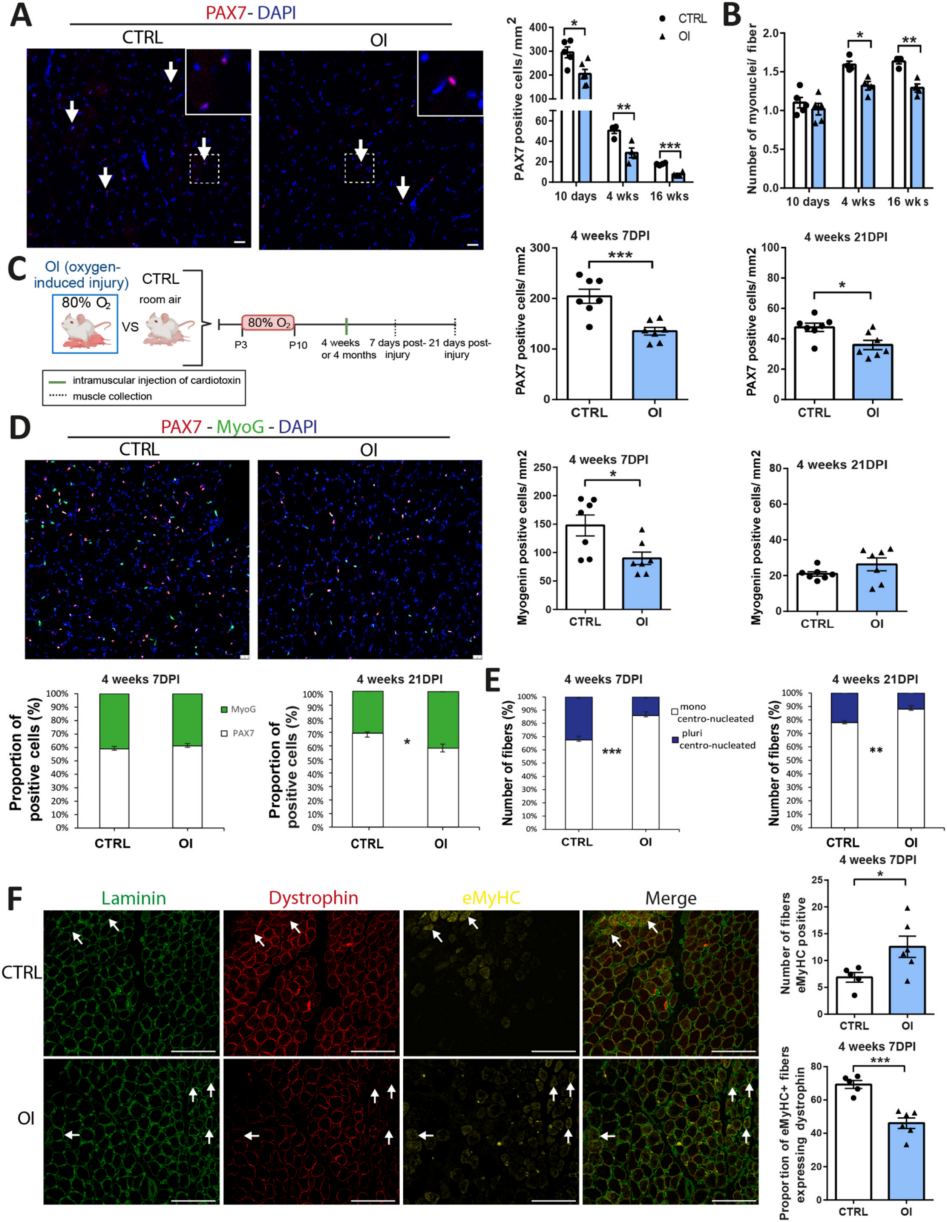
Impact of transient neonatal exposure to high oxygen on muscle stem cell pool and regenerative capacity. (A) Representative images of muscle stem cells (PAX7 positive, red) in the tibialis anterior (TA) sections from 4‐week‐old room air control (CTRL) and neonatal oxygen‐induced injury (OI) male rats. White arrows identify muscle stem cells. A magnified inset is shown in the top right corner. Density of PAX7 positive cells per mm^2^ and (B) number of myonuclei per fibre in the TA from CTRL versus OI males at 10 days, 4‐ and 16‐week postnatal. (C) Graphical representation of the experimental timeline created in BioRender.com. (D) Representative images of muscle stem cells (PAX7 positive, red) differentiated myoblasts (myogenin positive, green) and DAPI (nuclei, blue) in the TA sections from 4‐week CTRL and OI male rats at 7‐day postinjury (DPI). Density of PAX7 and myogenin positive cells per mm^2^, as well as the proportion of PAX7/myogenin cells in the TA from CTRL and OI male rats 7 and 21 DPI (injured at 4 weeks of age). (E) Proportion of fibres pluricentronucleated and monocentronucleated in the TA from 4‐week CTRL and OI male rats at 7 and 21 DPI. (F) Representative image of laminin (green), dystrophin (red) and embryonic myosin heavy chain (eMyHC) (yellow) staining of the TA from 4‐week CTRL and OI male rats at 7 DPI. Number of fibres eMyHC positive and proportion of eMyHC+ fibres positive for dystrophin in the TA from 4‐week CTRL and OI male rats at 7 DPI. White arrows identify immature regenerating myofibres expressing eMyHC and low/incomplete dystrophin expression. Number of fibres eMyHC positive and proportion of eMyHC+ fibres positive for dystrophin in the TA from 4‐week CTRL and OI male rats at 7 DPI. Error bars represent means ± SEM; (A, B) *n* = 4–6 per group; (D, E) *n* = 6–7 per group, (F) *n* = 5–6 per group. (A, D) Scale bars = 25 μm; (F) scale bars = 145 μm. Statistical analyses were performed using student *t* test to compare OI versus CTRL groups. *p < 0.05; **p < 0.01; ***p < 0.01 versus group indicated.

We used a rodent model of transient neonatal exposure to high oxygen (oxygen‐induced injury: OI), which is well established to mimic prematurity‐associated conditions such as bronchopulmonary dysplasia, retinopathy of prematurity, cardiovascular and skeletal muscle complications of adult and neonatal human tissues [10] (S20–S22). In OI versus CTRL males, PAX7 staining showed a reduction in the number of MuSCs per mm^2^ in the TA at 10 days (−30%, *p* = 0.0134), 4 weeks (−43%, *p* = 0.0075) and 16 weeks (−56%, *p* < 0.0001) in the OI groups (Figure 1A). A reduction in the number of nuclei per fibre, an indicator of myoblast fusion into myofibre, was also noted in the TA at 4 and 16 weeks (Figure 1B). In OI versus CTRL females, a similar but milder reduction in the number of MuSCs was observed (Figure S1).

To assess muscle regeneration, TA muscles were injured by an intramuscular injection of cardiotoxin and allowed to recover for seven (acute phase of regeneration) and 21 (return to homeostasis) days postinjury (DPI) (Figure 1C). In the TA of rats injured at 4 weeks of age, the number of MuSC per mm^2^ (PAX7‐positive cells) was reduced at seven and 21 DPI in the OI group compared to CTRL (Figure 1D). The number of differentiated myoblasts per mm^2^ (myogenin‐positive cells) was also decreased but only at seven DPI. The proportion of PAX7/myogenin cells was reduced at 21 DPI (Figure 1D), suggesting a delay in the return to homeostasis. The regenerating myofibres (centronucleated fibres) displayed a reduction in the number of pluricentronucleated fibres (> 2 centrally located nuclei per fibre) in the TA of OI males compared to CTRL at seven and 21 DPI, indicating reduced myonuclear accretion (i.e., addition of nuclei from MuSCs to muscle fibres) (Figure 1E). The evaluation of the number of fibres positive for embryonic Myosin Heavy Chain (eMyHC) revealed an increase in the number of regenerating myofibres in the OI group compared to CTRL (Figure 1F). To determine if this increase could be due to a delay in myofibre maturation, the eMyHC fibres were costained with dystrophin, a marker of mature myofibres (S24). Male OI group showed fewer mature fibres coexpressing eMyHC and dystrophin than CTRL (Figure 1F).

**FIGURE 2:**
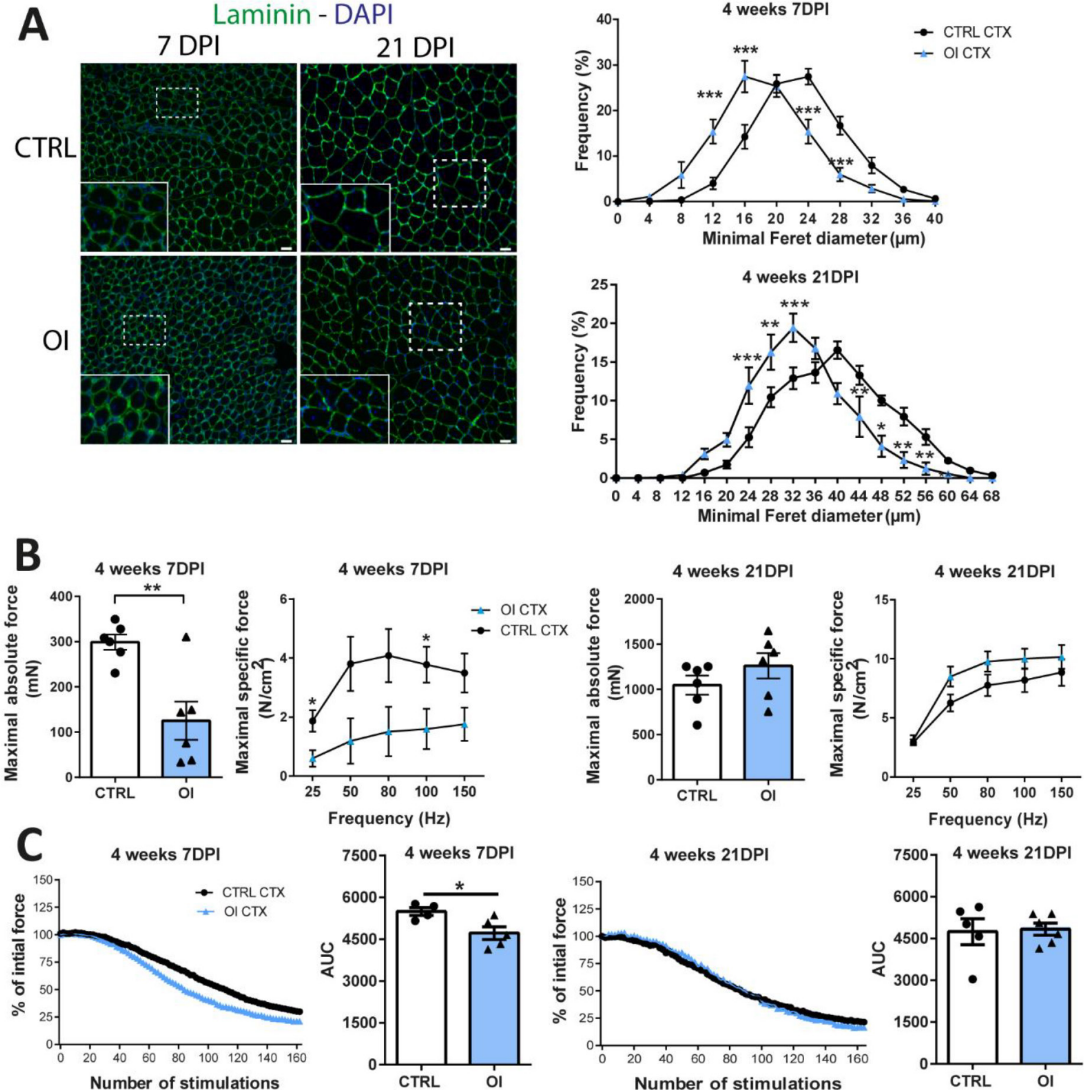
Impact of transient neonatal exposure to high oxygen on myofibre size and muscle contractile properties. (A) Representative images of myofibres stained with laminin (green) and DAPI (nuclei, blue) from tibialis anterior (TA) of 4‐week‐old room air control (CTRL) versus neonatal oxygen‐induced injury (OI) male rats. Minimal Feret's diameter distribution of centronucleated myofibres of the TA from 4‐week CTRL and OI male rats at 7‐ and 21‐day postinjury (DPI). (B) Maximal absolute force (mN, newton) and force‐frequency curve of the specific force (N/cm^2^); (C) fatigue curve and area under the curve (AUC) of the fatigue of the extensor digitorum longus (EDL) muscle from 4‐week‐old (CTRL) (OI) male rats at 7 and 21 DPI. Error bars represent means ± SEM; (A) *n* = 7 per group; (B) *n* = 6 per group and (C) *n* = 4–5 per group. (A) Scale bars = 25 μm. (A) Statistical analyses were performed using two‐way ANOVA**—**testing the effects of condition (OI vs. CTRL) and different diameter size—followed by Sidak post hoc test or (B, C) Student *t* test to compare OI versus CTRL groups. *p < 0.05; **p < 0.01; ***p < 0.001 versus group indicated.

To evaluate myofibre size, we measured the minimal Feret's diameter in 4‐week‐old male rats, which revealed a reduction in fibre calibre at seven and 21 DPI (Figure 2A). Evaluation of the contractile properties of the EDL muscle from OI male revealed lower force than CTRL for the absolute (−58%, *p* = 0.0034) and specific force (Figure 2B). There was no more significant difference between the groups at 21 DPI (Figure 2B). Repeated contractions showed higher fatigability in the EDL of OI male at seven DPI (AUC −14%, *p* = 0.0309), whereas no difference was observed at 21 DPI (Figure 2C).

Adult male rats injured at 16 weeks of age showed reduced MuSC pool, smaller myofibre size, lower absolute force, at a milder level than what is observed in juvenile rats injured at 4 weeks of age (Figures S2 and S3).

The females from the OI group presented regenerative defects similar to those observed in males when injured at 4 weeks of age, including reduced myogenic cell pool, reduced myonuclear accretion and smaller myofibres; however, these differences between OI and CTRL were largely no longer observed when injured at 16 weeks (Figures S1 and S4).

### 3.2 Impaired MuSC Fate Decision and Myogenic Progression After Neonatal Exposure to Preterm Birth‐Related Conditions

**FIGURE 3:**
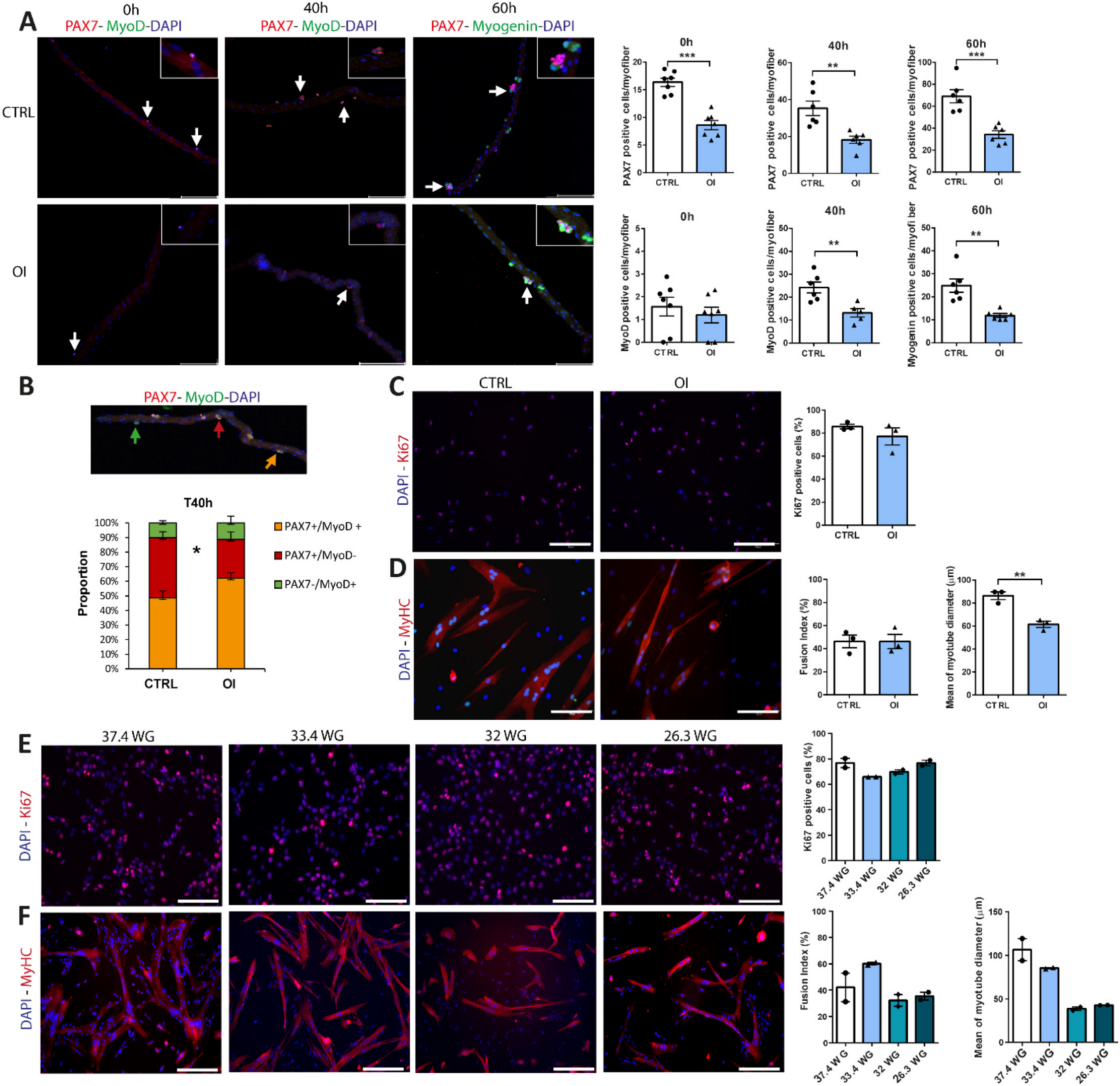
Impact of transient neonatal exposure to high oxygen and preterm birth on myogenesis capacity. (A, B) Single myofibres isolated from flexor digitorum brevis from 4‐week‐old room air control (CTRL) and neonatal oxygen‐induced injury (OI) male rats. (A) Representative images of single myofibres cultured for 0, 40 and 60 h to assess MuSC quiescence (PAX7 positive, red), activation (MyoD positive, green) and differentiation (Myogenin positive, green), respectively. White arrowheads indicate myogenic cells. Magnified insets are shown in top right corners. Numbers of PAX7 positive cells per myofibres at 0, 40 and 60 h, number of MyoD positive cells per myofibre at 0 and 40 h and number of myogenin positive cells per myofibre at 60 h. (B) Proportion of self‐renewing (Pax7 + MyoD−), proliferating (Pax7 + MyoD+) and differentiating (Pax7 − MyoD+) subsets of muscle stem cells in single myofibres cultured for 40 h. (C, D) Primary myoblasts isolated from 4‐week‐old CTRL and OI male rats. (C) Representative images of Ki67 (red) and DAPI (nuclei, blue) staining of myoblasts and quantification of the percentage of Ki67 positive cells in vitro. (D) Myosin heavy chain (MyHC positive, red) and DAPI (nuclei, blue) staining of myotubes in vitro and quantification of the fusion index and mean of myotube diameter (μm) in vitro. (E, F) Primary myoblasts isolated from deceased babies born preterm (26.3, 32 and 33.4 weeks of gestation, WG) and at term (37.4 WG). (E) Representative images of Ki67 (red) and DAPI (nuclei, blue) staining of myoblasts and quantification of the percentage of Ki67 positive cells in vitro. (F) MyHC (red) and DAPI (nuclei, blue) staining of myotubes in vitro and quantification of the fusion index and mean of myotube diameter (μm) in vitro. Error bars represent means ± SEM; (A, B) *n* = 6–7 independent biological samples per group; (C‐D) *n* = 3 independent biological samples per group; (E‐F) *n* = 2 technical replicates per independent biological sample. (A) Scale bars = 116 μm; (C, D) scale bars = 150 μm; (E, F) scale bars = 300 μm. Statistical analyses were performed using Student *t* test to compare OI vs CTRL groups. *p < 0.05; **p < 0.01; ***p < 0.001 versus group indicated.

To evaluate the myogenic capacity of the MuSC, we isolated and cultured single myofibres from the flexor digitorum brevis, a small foot muscle, of CTRL and OI male rats at 4 weeks of age. The number of MuSCs per myofibre is reduced in OI fibres at baseline (0 h), after their first cell division at 40 h and after multiple divisions at 60 h (Figure 3A). The number of MyoD (Myogenic Differentiation 1) (myoblasts) and myogenin (myocytes) positive cells was decreased in OI male at 40 and 60 h, respectively (Figure 3A). To assess if the cell fate decision of MuSC was also affected, the proportion of the different subpopulations—PAX7+/MyoD− (self‐renewing), PAX7+/MyoD+ (proliferative), PAX7−/MyoD+ (differentiating)—was assessed in myofibres cultured for 40 h. A specific reduction in the proportion of PAX7+/MyoD− was observed in the OI group, attesting to an alteration of the MuSC self‐renewal (Figure 3B).

To further investigate the myogenic progression of MuSCs, we sorted by FACS and cultured primary myoblasts from OI and CTRL male rats at 4 weeks of age. No difference in the proliferation capacity was observed between the groups (Figure 3C). After 3 days of differentiation, the fusion index was similar between the groups, but the mean myotube diameter was reduced (−28%, *p* = 0.0043) (Figure 3D).

To determine if similar changes could also be observed in humans born preterm, we collected muscle samples from deceased babies born at term or preterm. Similar to our rat model, the proliferative capacity of human myogenic cells did not seem to be affected (Figure 3E). However, samples from infants born at the lowest gestational ages (26.3 and 32 WG) showed a trend towards reduced myotube diameter (Figure 3F). Despite the limited number of human samples available, the results offer preliminary evidence that the myogenic cell alterations we identified in our animal models may also, at least in part, be recapitulated in cells from preterm‐born human infants.

### 3.3 Single‐Cell RNA‐Seq Identifies a Subset of MuSCs Exhibiting an Overactivation of the TNF‐α/NF‐κB Pathway After Transient Neonatal Exposure to High Oxygen

**FIGURE 4:**
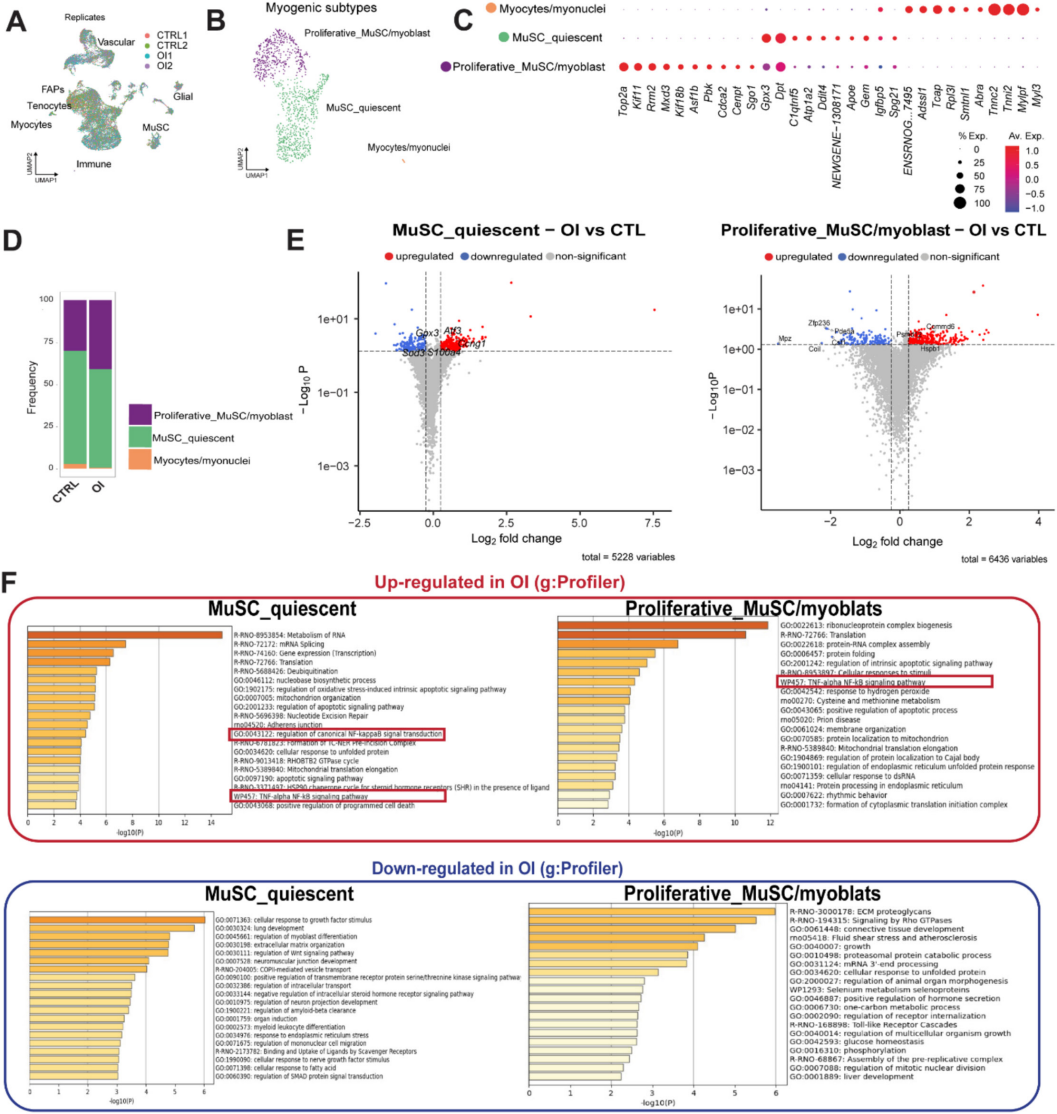
scRNA‐seq analysis revealed the molecular signature of subsets of muscle stem cells from control and oxygen‐induced injury rats. (A) UMAP embeddings displaying the room air control (CTRL) (red and green dots) and neonatal oxygen‐induced injury (OI) male rats cell populations (blue and purple dots) isolated at 4 weeks of age. (B) UMAP plot of pooled samples (CTRL and OI) showing the three different clusters of myogenic cells. (C) Dot plot diagram showing marker genes expressed in each cluster of myogenic cells. (D) Proportion of each cluster in CTRL versus OI male rats. (E) Volcano plots depicting differentially expressed genes (DEGs) downregulated or upregulated in MuSC quiescent or proliferative MuSC/myoblasts subsets from OI versus CTRL. (F) Functional enrichment analysis with GO Biological Processes analysis using DEGs downregulated and upregulated in MuSC quiescent or proliferative MuSC/myoblasts subsets from OI versus CTRL cells. The *X* axis represents the –log10(*p* value). *N* = 2 independent biological samples per group.

To define the molecular signature of MuSCs in OI versus CTRL conditions, scRNA‐seq was performed on the muscles of 4‐week‐old male rats (two CTRL and two OI), which were sorted (CD45‐, CD31‐ and CD11b‐) to obtain an enrichment in the proportion of MuSCs. After assignment, filtering and removal of doublets, we analysed 10 469 CTRL cells and 13 283 OI cells. The initial clustering resulted in seven clusters that each received a unique cell type annotation: MuSC, fibroadipogenic progenitors (FAPs), myocytes, tenocytes, vascular cells, glial cells and immune cells. The number of cells in each population, as well as uniform manifold approximations and projections (UMAPs), was comparable between conditions and biological replicates (Figures 4A and S5A,B). To further delineate the myogenic cells found in the dataset (1118 cells), this subset was set apart and reclustered into three clusters: MuSC_quiescent, Proliferative_MuSC/myoblast and myocytes/myonuclei (Figure 4B,C). We observed that the cell number was differently distributed between OI and CTRL, with more Proliferative_MuSC/myoblasts and fewer MuSC_quiescent and myocytes/myonuclei in OI (Figure 4D). Differential expression gene (DEGs) analysis showed that the expression of 506 genes was significantly changed (*p* < 10e−6; *n* = 174 downregulated and *n* = 332 upregulated) in the MuSC_quiescent cluster, 427 genes were significantly changed (*p* < 10e−6; *n* = 132 downregulated and *n* = 295 upregulated) in the MuSC/myoblast_proliferative cluster, and 37 genes were significantly changed (*p* < 10e−6; *n* = 2 downregulated and *n* = 35 upregulated) in the myocytes/myonuclei cluster (Figures 4E and S5D) from OI compared to CTRL. Gene Ontology analysis of MuSC_quiescent and Proliferative_MuSC/myoblasts revealed that downregulated genes in OI cells are associated with biological processes related to cell cycle, myoblast differentiation, cellular response to growth factor and extracellular matrix organization. Upregulated genes in OI cells were enriched for TNF‐α/NF‐κB signalling pathway, apoptosis, RNA translation, mitochondria organization and response to hyperoxia (Figure 4F).

**FIGURE 5:**
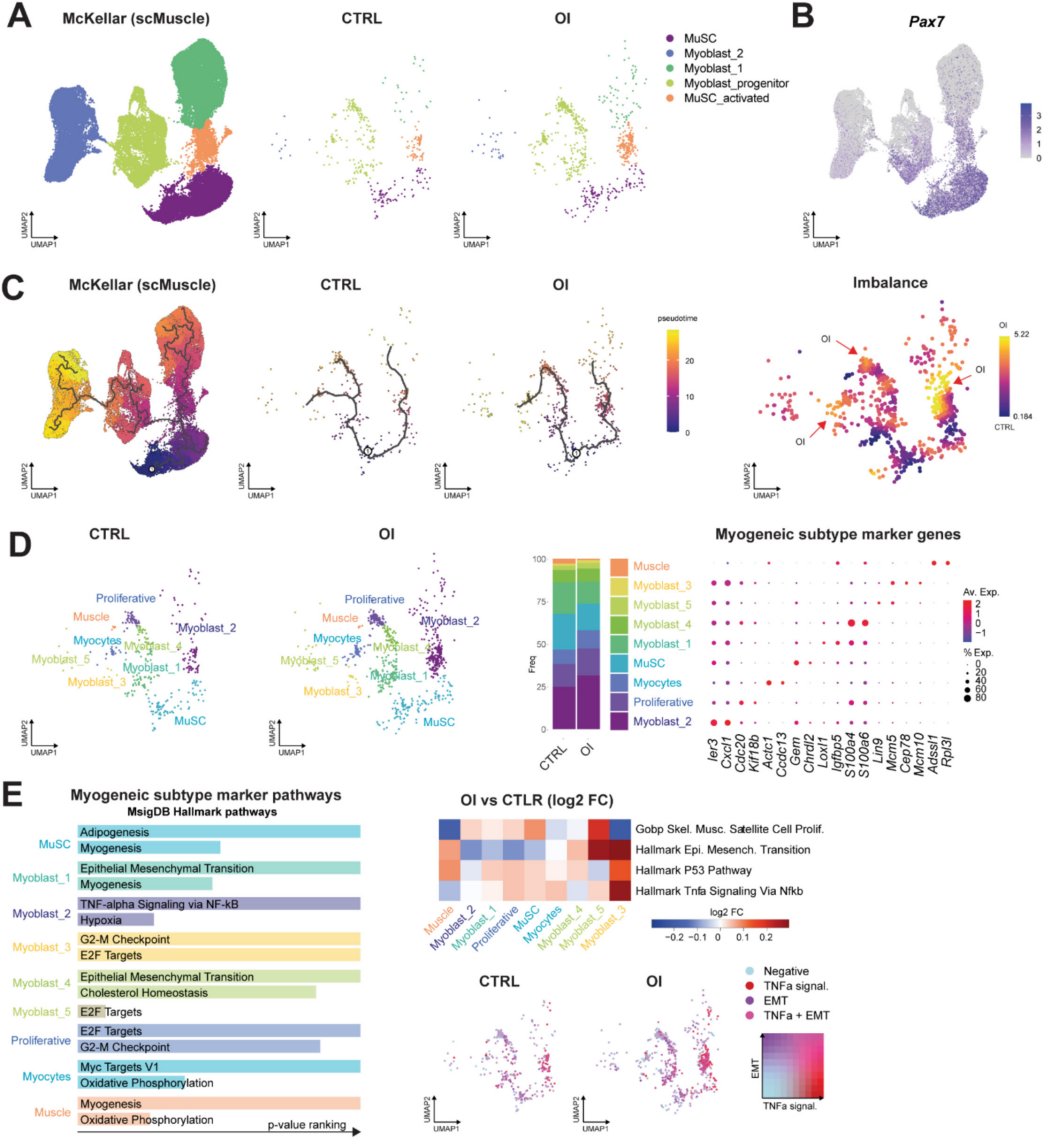
Identification via scRNA‐seq analysis of muscle stem cell trajectory after transient neonatal exposure to high oxygen. (A) UMAP plot of the dataset from McKellar et al. [15], as well as room air control (CTRL) and neonatal oxygen‐induced injury (OI) male rats at 4 weeks after alignment with McKellar et al. [15] to refine the clustering. (B) UMAP plot of pooled datasets (CTRL and OI and McKellar) showing *Pax7* gene expression. (C) Trajectory analysis of MuSC differentiation in McKellar et al. [15] dataset, CTRL and OI male rat cells. (D) UMAP embeddings showing the nine different clusters of myogenic cells in CTRL and OI male rat samples. Proportion of the nine clusters in CTRL versus OI male rats and dot plot diagram representation showing markers genes differentially expressed in each cluster of myogenic cells. (E) Functional enrichment analysis with GO biological processes analysis using genes upregulated for each cluster. Heatmap showing the fold change of GSVA score on gene sets related to TNFα NF‐κB signalling pathway, epithelial mesenchymal transition (EMT), muscle stem cells proliferation and p53 pathways comparing DEGs transcripts from each cluster. UMAP plot shows cells with positive GSVA values for all the gene sets were annotated as TNFα + EMT, whereas clusters with only one gene set were annotated TNFα or EMT. Negative GSVA scores represents cells negative for both gene sets. *n* = 2 independent biological samples per group.

Next, we performed a pseudotime analysis to infer the progression of myogenic cells by ordering them along a trajectory defined by gradual changes in their gene‐expression profiles. To improve clustering accuracy, we aligned our data set with publicly available skeletal muscle scRNA‐seq (McKellar et al. [15]) (Figure 5A,B). This revealed divergent trajectory paths in OI versus CTRL cells, highlighting three imbalance points (Figure 5C). To capture these differences, we then reclustered the combined data based on the trajectory‐associated gene expression. This resulted in nine clusters that each received a unique cell type annotation: MuSC (quiescent), proliferative MuSC, myoblast_1, myoblast_2 (immunomyoblasts), myoblast_3, myoblast_4, myoblast_5, myocytes and myonuclei (myofibres) (Figure 5D). The imbalance spots observed in the pseudotime analysis corresponded to the myoblast_2 (immunomyoblasts), proliferative and myocytes clusters (Figure 5C,D). We observed that the cell number was differently distributed between groups, with more myoblast_2 (enriched in TNF‐α signalling via NF‐kB), and less MuSC (quiescence) in the OI group (Figure 5E). Using heatmap representation, we show that this enrichment of the TNF‐α/NF‐κB pathway is observed in various clusters in the OI condition along the trajectory (Figure 5E).

**FIGURE 6:**
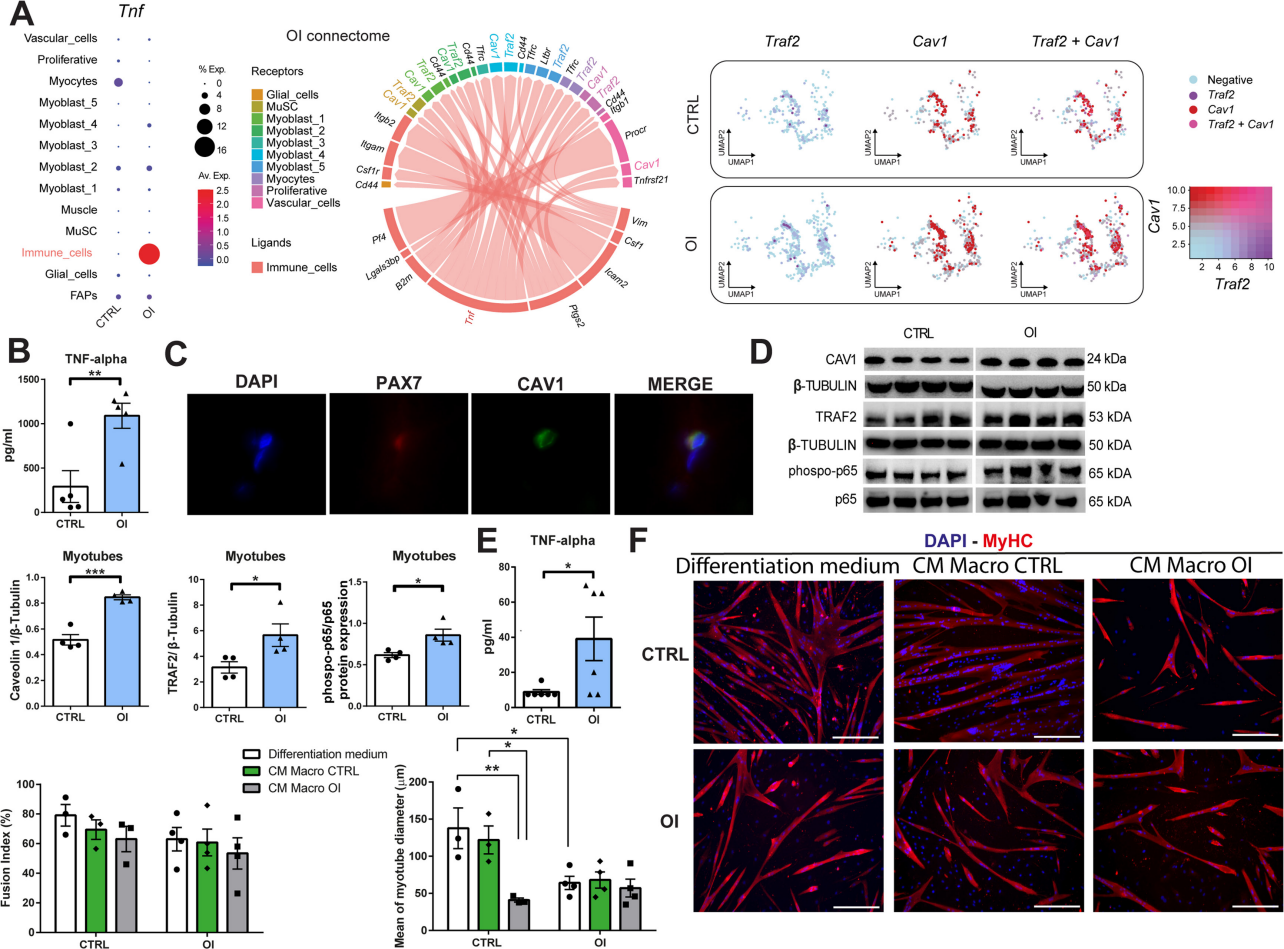
Identification via scRNA‐seq analysis of cell–cell interaction after transient neonatal exposure to high oxygen. (A) Dot plot representing gene expression of *Tnf* in each cell type from CTRL and OI male rat cells. Connectome analysis between *Tnf* from OI immune cells and the nine different clusters of myogenic cells and other cell types. UMAP plot representation of gene expression of *Cav1* and *Traf2* in CTRL and OI. UMAP plot showing cells with positive GSVA values for all the gene sets were annotated as *Traf2* + *Cav1*, whereas clusters with only one gene set were annotated Traf2 or Cav1. Negative GSVA scores represent cells negative for both gene sets. (B) Protein levels of TNF‐α in tibialis anterior from male CTRL and OI at 4 weeks. (C) Representative images of coexpression by immunostaining of PAX7 (quiescent MuSC; red) and Caveolin 1 (CAV1; green). (D) Representative images of Western blots showing the protein expression and the ratio of relative protein expression of Caveolin1 (CAV1), TNF receptor‐associated factor 2 (TRAF2), phospho‐p65, p65 and β‐tubulin (as loading control) from in vitro myotubes of 4‐week‐old CTRL vs OI male rats. (E) Protein levels of TNF‐α in macrophage medium conditioned from male CTRL and OI. (F) MyHC staining (red) of myotubes in vitro and quantification of the fusion index and mean of myotube diameter (μm) in vitro of muscle stem cells isolated and cultured either with differentiation medium or with CTRL or OI macrophage conditioned medium (CM). Error bars represent means ± SEM; (A) *n* = 2 independent biological samples per group; (B) *n* = 5 independent biological samples per group; (D) *n* = 4 independent biological samples per group; (E) *n* = 6 independent biological samples per group; (F) *n* = 3–4 independent biological samples per group. (F) Scale bars = 300 μm. (B, D, E) Statistical analyses were performed using Student *t* test to compare OI versus CTRL groups or (F) two‐way ANOVA—testing the effects of condition (OI vs. CTRL) and treatment (differentiation medium vs. CM Macro CTRL vs. CM Macro OI) and their interaction—followed by Tukey's post hoc test. *p < 0.05; **p < 0.01 vs. group indicated.

To determine the source of the TNF‐α signalling in OI cells, we assessed its expression in all cell types in our dataset. In OI male rats, immune cells displayed the highest expression level of TNF‐α (Figure 6A). To assess the impact of paracrine factors secreted by immune cells on myogenic cells, we performed a cell–cell communication (connectome) analysis. We observed that the *Tnf* ligand expression by immune cells had a strong interaction with *Traf2* (TNF receptor‐associated factor 2) and *Cav1* (Caveolin 1) genes that were upregulated in OI myogenic cells (Figure 6A).

Using an ELISA assay, we validated the increase in TNF‐α protein levels (*p* = 0.0080) in the TA from OI versus CTRL rats (Figure 6B). The protein expression of CAV1 by the MuSC (PAX7 positive cells) was confirmed by immunofluorescence on muscle sections in vivo (Figure 6C). Further analysis of CAV1, TRAF2 and NF‐κB (phospho‐p65/p65) protein expression revealed upregulated expression in myotubes from OI rats compared to CTRL (Figure 6D). Considering the myotube atrophy phenotype, we also evaluated atrophy‐related proteins and observed an increased expression of MuRF‐1 but no difference for atrogin‐1 expression in OI versus CTRL males (Figure S6A,B). In humans, higher protein expression levels of the phospho‐p65/p65 ratio were observed for the two infant males born preterm compared to the male infant born at term and the female infant born preterm (Figure S6C).

To further investigate the macrophage and MuSC interactions, we isolated both cell populations from the muscles of 4‐week‐old OI and CTRL male rats by FACS and cultured them in vitro. Conditioned medium from macrophages was collected, and the increase in TNF‐α protein levels in the OI samples was confirmed by ELISA (Figure 6E). Myoblasts were differentiated with conditioned macrophage medium for 48 h, which revealed a reduction in the myotube diameter in CTRL myotubes treated with conditioned macrophage medium from OI male rats compared to the one from CTRL (−66%, *p* = 0.0216) (Figure 6F).

### 3.4 TNF‐α Inhibitor Improves MuSC Regenerative Capacity and Mitigates the Oxygen‐Induced Myopathy

**FIGURE 7:**
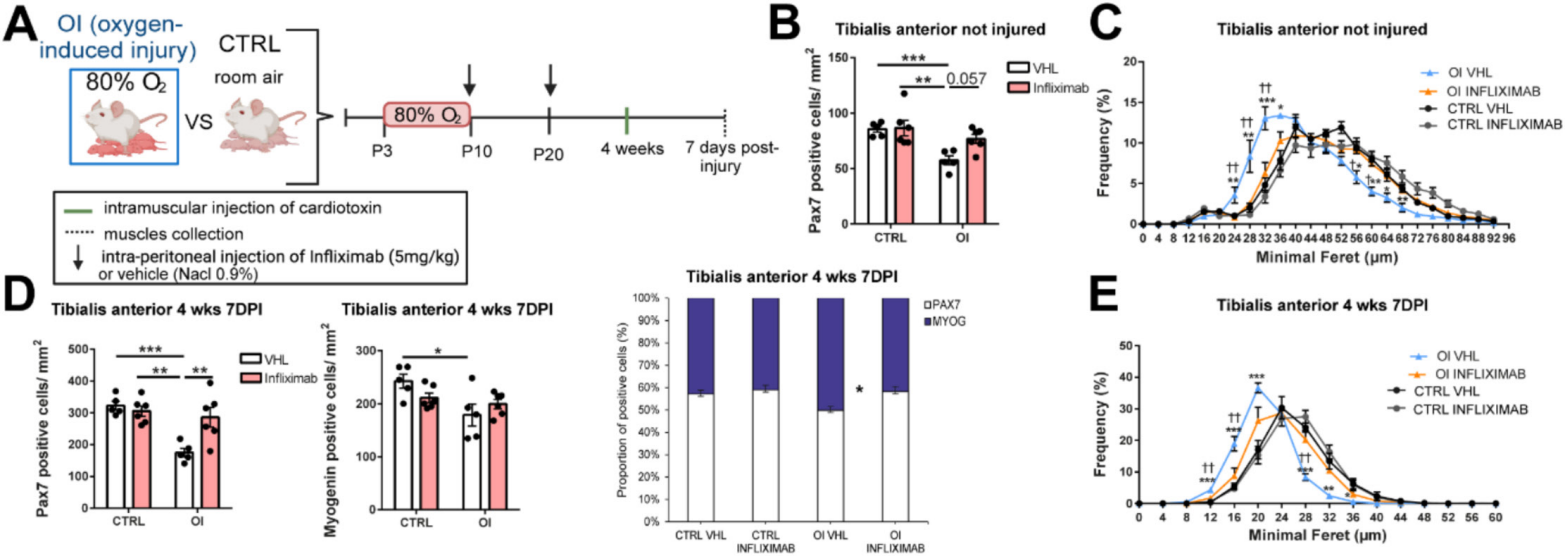
TNF‐α inhibitor restores the oxygen‐induced myopathy and the myogenesis capacity. (A) Graphical representation, created in Biorender.com, of the experimental design. (B) Number of PAX7 positive cells per mm^2^ and (C) minimal Feret's diameter distribution in uninjured tibialis anterior (TA) muscle from 4‐week‐old room air control (CTRL) versus neonatal oxygen‐induced injury (OI) male rats treated with Infliximab or vehicle (VHL). (D) Number of PAX7 positive cells per mm^2^, myogenin positive cells per mm^2^ and proportion of monocentronucleated versus pluricentronucleated fibres in the TA of 4‐week‐old male rats at 7‐day postinjury (DPI). (E) Minimal Feret's diameter of centronucleated myofibres of the TA from 4‐week‐old CTRL VHL, OI VHL, CTRL infliximab and OI infliximab male rats at 7 DPI. Error bars represent means ± SEM; *n* = 6 independent biological samples per group for CTRL and OI infliximab; *n* = 5 for CTRL and OI VHL. Statistical analyses were conducted by two‐way ANOVA—testing the effects of condition (OI vs. CTRL) and treatment (infliximab vs. vehicle) and their interaction—followed by Tukey's post hoc test. *p < 0.05, **p < 0.01, ***p < 0.001 versus group indicated. (C, E) *OI VHL versus CTRL groups; ^†^OI VHL versus OI infliximab.

Next, we aimed to determine if an inhibitor of TNF‐α (Infliximab) could restore MuSC function exposed to preterm birth‐related conditions. After transient neonatal exposure to high oxygen level, rats received either 5 mg/kg of Infliximab i.p. or vehicle (VHL; NaCl 0.9%) at 10 and 20 days of life (Figure 7A). No difference in body weight was observed between the groups (Figure S7A). Infliximab treatment did not affect the MuSC count in the CTRL group, but in the OI‐treated group, it showed a strong trend towards an increased MuSC pool compared to OI VHL, although this did not reach statistical significance (*p* = 0.057) (Figure 7B). Moreover, Infliximab increased the size of the fibres in the OI group, which was restored to the level of CTRL groups (Figure 7C).

To assess the effect of Infliximab on muscle regeneration, muscle injury was induced by an intramuscular injection of cardiotoxin at 4 weeks; muscles were collected at 7 DPI (Figure 7A). The ratio of TA muscle to body weight was reduced in OI VHL compared to the CTRL groups, but Infliximab treatment in the OI group restored muscle weight to the level of CTRL groups (Figure S7C). Infliximab treatment restored the number of MuSCs in the injured OI group (*p* = 0.0073 OI VHL vs. infliximab), whereas it had no effect on the CTRL group (Figure 7D). However, Infliximab treatment had no effect on the number of myogenin cells in the OI group. The proportion of pluricentronucleated fibres and the size of the regenerating fibres was rescued by Infliximab in the OI group to the level of CTRL (Figure 7D,E).

## 4 Discussion

Genetic mutations leading to different myopathies were shown to impair MuSC function and contribute to disease progression, which led to the emergence of a class of satellite cell‐opathies [16] (S24, S25). Accumulating evidence also indicates that the microenvironment, such as inflammation and oxygen levels, could affect the MuSC function in various acquired myopathies [17]. In chronic diseases, such as chronic obstructive pulmonary disease and heart failure, it was found that low‐grade inflammation impairs myogenic capacity and therefore contributes to the progression of muscle atrophy and the diseases [17] (S26–S30). In ageing, an accumulation of senescent cells creates an aged‐like inflamed niche that impairs MuSC regenerative capacity [14]. Our findings indicate that transient hyperoxic stress during the critical neonatal period promotes a long‐lasting proinflammatory microenvironment that contributes to the exhaustion of the MuSC pool associated with impaired self‐renewal and regenerative capacity at juvenile and adult stages.

Our previous findings indicated that there is no muscle atrophy immediately after the hyperoxic period at 10 days postnatal but that the muscle dysfunction appears over time (e.g., increased MuRF‐1 and atrogin‐1 protein expression levels as well as collagen type I deposition), suggesting developmental delay [10]. Our new findings show that MuSCs are rapidly affected after the transient neonatal hyperoxia period, suggesting that MuSC defects are an upstream event that is at the root cause of the muscle wasting and weakness observed in individuals born preterm [11]. These findings are coherent with other studies showing the key role played by MuSCs in postnatal muscle development and growth [18, 19].

The alteration of stem cell potential after exposure to preterm birth‐related conditions was reported in other organs/systems such as lungs [20] and blood vessels [21]. Adult mice exposed to neonatal high levels of oxygen have fewer alveolar type II cells [22] (considered as the lung cells with high stem cell potential [S31]) and impaired differentiation into type I cells [20] affecting the recovery and proper development of the lungs [23]. In adult humans born preterm, the endothelial progenitor cells have a reduced proliferation and vasculogenesis capacity [21]. These findings support that stem cell defects are a hallmark of preterm birth‐related conditions [24].

Progenitor cell stemness maintenance, proliferation or differentiation rely in part on mitochondria, which are sensitive to oxygen tension [25] (S31), suggesting that a dysfunction of these organelles could underlie the MuSC alteration. Supporting this, our recent work demonstrated mitochondrial impairments in cardiomyocytes related to cardiac changes in OI male rats (S22).

Single‐cell transcriptomic analysis identified upregulation of the TNF‐α/NF‐κB signalling pathway in subsets of MuSCs, which is consistent with our previous study showing a higher level of NF‐κB in the whole TA muscle of juvenile male rats [10]. In MuSCs, TNF‐α activates the canonical NF‐κB pathway, which stimulates cell proliferation through cyclin‐D1 but inhibits MyoD and myogenin expression, resulting in impaired differentiation [26] (S33, S34). Connectome analysis revealed that *Tnf* was highly expressed by immune cells in OI rats, which interact with *Traf2* and *Cav1* expressed by MuSCs. CAV1 and TRAF2 interact together to form a complex with the TNF‐receptor to regulate its distribution and activation of NF‐κB [27]. CAV1 is highly expressed in proliferating myoblasts [28] (S35), and it plays a role in the regulation of MuSC proliferation and differentiation [28, 29] (S36, S37). In transgenic mice overexpressing CAV1, the skeletal muscle tissue fails to repair after a cardiotoxin injury [29]. A dysregulation of CAV1 associated with enhanced proinflammatory cytokines such as TNF‐α is observed in chronic conditions such as diabetes and obesity [30] (S38, S39). These findings are consistent with the high CAV1 expression observed all along the trajectory of OI myoblasts and their impaired myogenic progression.

TNF‐α signalling was shown to affect muscle cells through redox imbalance [31], activation of mitophagy [32] and mitochondrial fragmentation [33]. These findings are coherent with results from another group showing signs of mitochondrial damage and lower mitochondrial oxidative capacity in skeletal muscle of adult rats exposed to transient neonatal hyperoxia (S20). Further studies are required to elucidate the TNFα–NF‐κB–mitochondrial dysregulation cascade and its consequences for MuSC function under preterm birth‐associated conditions.

Increased circulating inflammatory markers [34] and activation of the NF‐κB signalling [35] are also observed in ageing, contributing to a reduced MuSC pool and alterations in skeletal muscle mass and function that promote muscle frailty and reduced mobility [35] (S40). Transplantation of wild‐type bone marrow cells into TNF‐α‐null mice demonstrated that the TNF‐α secreted by myeloid cells contributes to the MuSC defect and muscle wasting in aged mice [36]. Another study showed that the administration of an NF‐κB inhibitor restores the lost function of MuSC due to ageing [35]. Taken together, these findings suggest that the MuSC defect induced by preterm‐birth‐related conditions resembles accelerated ageing.

Our findings demonstrated that inhibition of TNF‐α using Infliximab restores the MuSC pool in uninjured muscles and their regenerative potential postinjury. Infliximab is well established as a safe therapy for paediatric patients with acute severe ulcerative colitis (S41) and Crohn's disease (S42). In adult and paediatric patients with Crohn's disease, studies reported that anti‐TNF‐α therapy reverses signs of inflammatory‐induced muscle wasting [37] (S43). TNF‐α blockade by another drug, etanercept, also showed a protective effect on muscle wasting in aged mice [38]. Although many elements need to be taken into consideration when administering drugs as a first line for infants born preterm, our findings provide a proof of concept that the detrimental effect of preterm birth associated conditions on MuSCs could be reversed therapeutically.

This study has some limitations and alternative explanations to consider. Although the transient exposure to neonatal hyperoxia is extensively used to study short‐ and long‐term complications of preterm birth [10] (S20, S22, S44), it does not fully recapitulate the complexity of preterm birth‐related conditions. For instance, prematurity‐associated complications and treatments such as parenteral nutrition and corticosteroids could also contribute to disrupting postnatal muscle growth. Nevertheless, our previous findings demonstrated that the model is relevant to investigate the impact of preterm‐birth related conditions in skeletal muscle, considering that the maturational stage of skeletal muscle in 3‐day‐old rats corresponds to that of a human infant born very preterm [10]. Moreover, data from human myogenic cells comparing preterm and term infants exhibited similar trends to those observed in our experimental model, although validation in larger cohorts will be necessary. Another limitation of this experiment is the lack of a sample from term‐born females. However, because females are generally less susceptible to both the short‐ and long‐term complications of preterm birth [39, 40], including term‐born female data would likely have accentuated the differences observed between preterm and term groups.

In summary, preterm birth‐related conditions trigger the TNF‐α/NF‐kB pathway in MuSCs, thereby impacting their cell fate decision and regenerative capacity. These alterations can lead to long‐lasting changes in muscular health, which can be reversed by early life treatment with an inhibitor of TNF‐α. Considering the vital role skeletal muscle plays in the quality of life and the development of chronic illnesses, it is imperative to incorporate efforts to enhance skeletal muscle health for individuals born prematurely, who face heightened risks of cardiovascular and metabolic conditions. Furthermore, with the significant increase in the survival rates of preterm born individuals over the past decades, a large cohort of individuals is now entering adulthood and will encounter skeletal muscle‐related issues associated with accelerated ageing. This study addresses the urgent need for a better understanding and treatment of these dysfunctions.

## Conflicts of Interest

The authors declare no conflicts of interest.

## Supporting information


**Figure S1:** Impact of transient neonatal exposure to high oxygen on muscle stem cell pool and regenerative capacity at 4 weeks in female rats. (A) Density of PAX7 positive cells per mm^2^ in the tibialis anterior (TA) from room air control (CTRL) and neonatal oxygen‐induced injury (OI) female rats at 10 days, 4‐ and 16 weeks (wks). Animals from the neonatal oxygen‐induced injury (OI) or room air control (CTRL) groups were injured, and muscle regeneration was assessed at 7‐ and 21 days post‐injury (DPI). (B) Density of muscle stem cells (PAX7 positive) and differentiated myoblasts (Myogenin positive) per mm^2^, and the proportion of PAX7/Myogenin cells in the TA from 4 wks old female rats at 7‐ and 21 days post‐injury (DPI). (C) Proportion of pluri‐ and mono‐centronucleated fibres in the TA from 4 wks CTRL and OI female rats at 7‐ and 21 DPI. (D) Minimal Feret's diameter of centronucleated myofibers of the TA from 4 wks old CTRL and OI female rats at 7‐ and 21 DPI. Error bars represent means ± SEM; (A) *n* = 4–6, and (B‐K) *n* = 6–7 per group. (A‐C) Statistical analyses were performed using student t‐test to compare OI vs CTRL or (D) two‐way ANOVA—testing the effects of condition (OI vs. CTRL) and different diameter size—followed by Sidak post hoc test. *p < 0.05; **p < 0.01; *** *p* < 0.001 vs. group indicated.
**Figure S2:** Impact of transient neonatal exposure to high oxygen on muscle regeneration capacity in 16‐weeks‐old male rats. (A) Number of muscle stem cells (PAX7‐positive) differentiated myoblasts (Myogenin‐positive) per mm^2^, and proportion of PAX7 and Myogenin positive cells in the tibialis anterior (TA) of 16 weeks (wks) old male rats at 7‐ and 21 days post‐injury (DPI). (B) Proportion of pluri‐ and mono‐centronucleated fibres in the TA of 16 wks CTRL and OI male rats at 7‐ and 21 DPI. (C) Minimal Feret's diameter of centronucleated myofibers in the TA of 16 wks CTRL and OI male rats at 7‐ and 21 DPI. Error bars represent means ± SEM; *n* = 5–6 per group. (A‐B) Statistical analyses were performed using student t‐test to compare OI vs CTRL or (C) two‐way ANOVA—testing the effects of condition (OI vs. CTRL) and different diameter size—followed by Sidak post hoc test. *p < 0.05; **p < 0.01 vs. group indicated.
**Figure S3:** Impact of transient neonatal exposure to high oxygen on muscle contractile properties at 16 weeks in male rats. (A,B) Maximal specific force, and force‐frequency curve of the extensor digitorum longus (EDL) muscle from 16 weeks (wks) old room air control (CTRL) vs. neonatal oxygen‐induced injury (OI) male rats at 7‐ and 21 days post‐injury (DPI). Error bars represent means ± SEM; *n* = 5–6 per group. Statistical analyses were performed using student t‐test to compare OI vs CTRL groups.
**Figure S4:** Impact of transient neonatal exposure to high oxygen on muscle regeneration capacity in 16 weeks old female rats. (A) Number of muscle stem cells (PAX7 positive) differentiated myoblasts (Myogenin positive) per mm^2^, as well as the proportion of PAX7/Myogenin cells in the tibialis anterior (TA) of 16 weeks (wks) old room air control (CTRL) and neonatal oxygen‐induced injury (OI) female rats at 7‐ and 21 days post‐injury (DPI). (B) Proportion of pluri‐ and mono‐centronucleated fibers in the TA from 16 wks CTRL and OI female rats at 7‐ and 21 DPI. (C) Minimal Feret's diameter of centronucleated myofibers of the TA from 16 wks CTRL and OI female rats at 7‐ and 21 DPI. Error bars represent means ± SEM; *n* = 5–6 per group. (A‐B) Statistical analyses were performed using student t‐test to compare OI vs CTRL or (C) two‐way ANOVA—testing the effects of condition (OI vs. CTRL) and different diameter size—followed by Sidak post hoc test. *p < 0.05; **p < 0.01 vs. group indicated.
**Figure S5:** scRNA‐seq analysis of muscle stem cells from control and oxygen‐induced injury male rats. (A) UMAP of the room air control (CTRL) and neonatal oxygen‐induced injury (OI) male rats cell populations at 4 weeks. (B) Dot plot diagram showing markers genes expressed in each cluster of cells population. (C) UMAP embeddings CTRL and OI male rat cells displaying gene expression of *PAX7*. (D) Volcano plot depicting differentially expressed genes (DEGs) downregulated or upregulated in OI cells vs. CTRL myocytes/myonuclei subset. (E) Functional enrichment analysis with GO Biological Processes analysis using DEGs downregulated and upregulated in OI vs. CTRL cells. The X‐axis represents the –log10(P‐value). *N* = 2 independent biological samples/group.
**Figure S6:** Quantification by western blot of Caveolin 1, TRAF2, atrogenes and NF‐kB protein. (A) Images of the full unedited western blots showing the protein expression of Caveolin1, TRAF2, phospho‐p65, p65 and β‐Tubulin (as loading control) from in vitro myotubes of 4 weeks old CTRL and OI male rats. (B) Images of the full unedited Western blots and quantification of the protein expression, Muscle RING Finger 1(MuRF‐1), Atrogin‐1, phospho‐p38 MAPK, p38 MAPK and β‐Tubulin (as loading control) from in vitro myotubes of 4 weeks old CTRL and OI male rats. (C) Images of the full unedited Western blots and quantification of the protein expression of phospho‐p65, p65 from in vitro myotubes isolated from deceased babies born preterm (26.3, 32 and 33.4 weeks of gestation (WG) and at term (37.4 WG).
**Figure S7:** TNF‐α inhibitor impact on the body weight and muscle weight of juvenile male rats. (A) Body weight (g) of room air control (CTRL) vs. neonatal oxygen‐induced injury (OI) male rats treated with infliximab or vehicle (VHL). Ratio of muscle weight (mg) on body weight (g) from (B) uninjured tibialis anterior (TA) at 4 weeks (wks) and (C) TA after 7 days post‐injury (DPI). (D) proportion of PAX7 and Myogenin positive cells of the TA from CTRL vs. OI male rats at 4 wks of age and 7 DPI treated with infliximab or VHL. Error bars represent means ± SEM; *n* = 6 independent biological sample per group for CTRL and OI infliximab; *n* = 5 for CTRL and OI VHL. Statistical analyses were performed using Two‐way ANOVA —testing the effects of condition (OI vs. CTRL) and treatment (VHL vs. Infliximab) and their interaction—followed by Tukey's post hoc test. *p < 0.05 vs. group indicated.

**Data S1:** Supplementary Information

## Data Availability

All data needed to evaluate the conclusions in the paper are present in the paper and/or the Supporting Information. Single‐cell RNA seq data have been deposited to Gene Expression Omnibus under Accession Number GSE271974. Data are available for sharing upon reasonable requests to the corresponding authors.
