# Supplementary material for: Preterm Birth Conditions Alter Muscle Stem Cells and Their Niche, Causing Lasting Impairments in Muscle Regeneration and Function
Source: J Cachexia Sarcopenia Muscle. 2025 Sep 16;16(5):e70058. doi: 10.1002/jcsm.70058 (PMC12439181; doi:10.1002/jcsm.70058)
Supplement: Supplementary file 1 — Figure S1: Impact of transient neonatal exposure to high oxygen on muscle stem cell pool and regenerative capacity at 4 weeks in female rats. (A) Density of PAX7 positive cells per mm2 in the tibialis anterior (TA) from room air control (CTRL) and neonatal oxygen‐induced injury (OI) female rats at 10 days, 4‐ and 16 weeks (wks). Animals from the neonatal oxygen‐induced injury (OI) or room air control (CTRL) groups were injured, and muscle regeneration was assessed at 7‐ and 21 days post‐injury (DPI). (B) Density of muscle stem cells (PAX7 positive) and differentiated myoblasts (Myogenin positive) per mm2, and the proportion of PAX7/Myogenin cells in the TA from 4 wks old female rats at 7‐ and 21 days post‐injury (DPI). (C) Proportion of pluri‐ and mono‐centronucleated fibres in the TA from 4 wks CTRL and OI female rats at 7‐ and 21 DPI. (D) Minimal Feret's diameter of centronucleated myofibers of the TA from 4 wks old CTRL and OI female rats at 7‐ and 21 DPI. Error bars represent means ± SEM; (A) n = 4–6, and (B‐K) n = 6–7 per group. (A‐C) Statistical analyses were performed using student t‐test to compare OI vs CTRL or (D) two‐way ANOVA—testing the effects of condition (OI vs. CTRL) and different diameter size—followed by Sidak post hoc test. *p < 0.05; **p < 0.01; *** p < 0.001 vs. group indicated. Figure S2: Impact of transient neonatal exposure to high oxygen on muscle regeneration capacity in 16‐weeks‐old male rats. (A) Number of muscle stem cells (PAX7‐positive) differentiated myoblasts (Myogenin‐positive) per mm2, and proportion of PAX7 and Myogenin positive cells in the tibialis anterior (TA) of 16 weeks (wks) old male rats at 7‐ and 21 days post‐injury (DPI). (B) Proportion of pluri‐ and mono‐centronucleated fibres in the TA of 16 wks CTRL and OI male rats at 7‐ and 21 DPI. (C) Minimal Feret's diameter of centronucleated myofibers in the TA of 16 wks CTRL and OI male rats at 7‐ and 21 DPI. Error bars represent means ± SEM; n = 5–6 per group. (A‐B) Statistic [file JCSM-16-e70058-s001.docx]

**Supplemental Figures for**

**Preterm birth conditions alter muscle stem cells and their niche, causing lasting impairments in muscle regeneration and function.**

Alyson Deprez^1,2^, Thomas Molina^1,2^, Gael Cagnone^1^, Pauline Garcia^1^, Séverine Leclerc^1^, Anik Cloutier^1^, Rebecca Desaulniers^1,2^, Benjamin Ellezam^1^, Anne Monique Nuyt^1,3*^, Nicolas A. Dumont^1,4*^

^
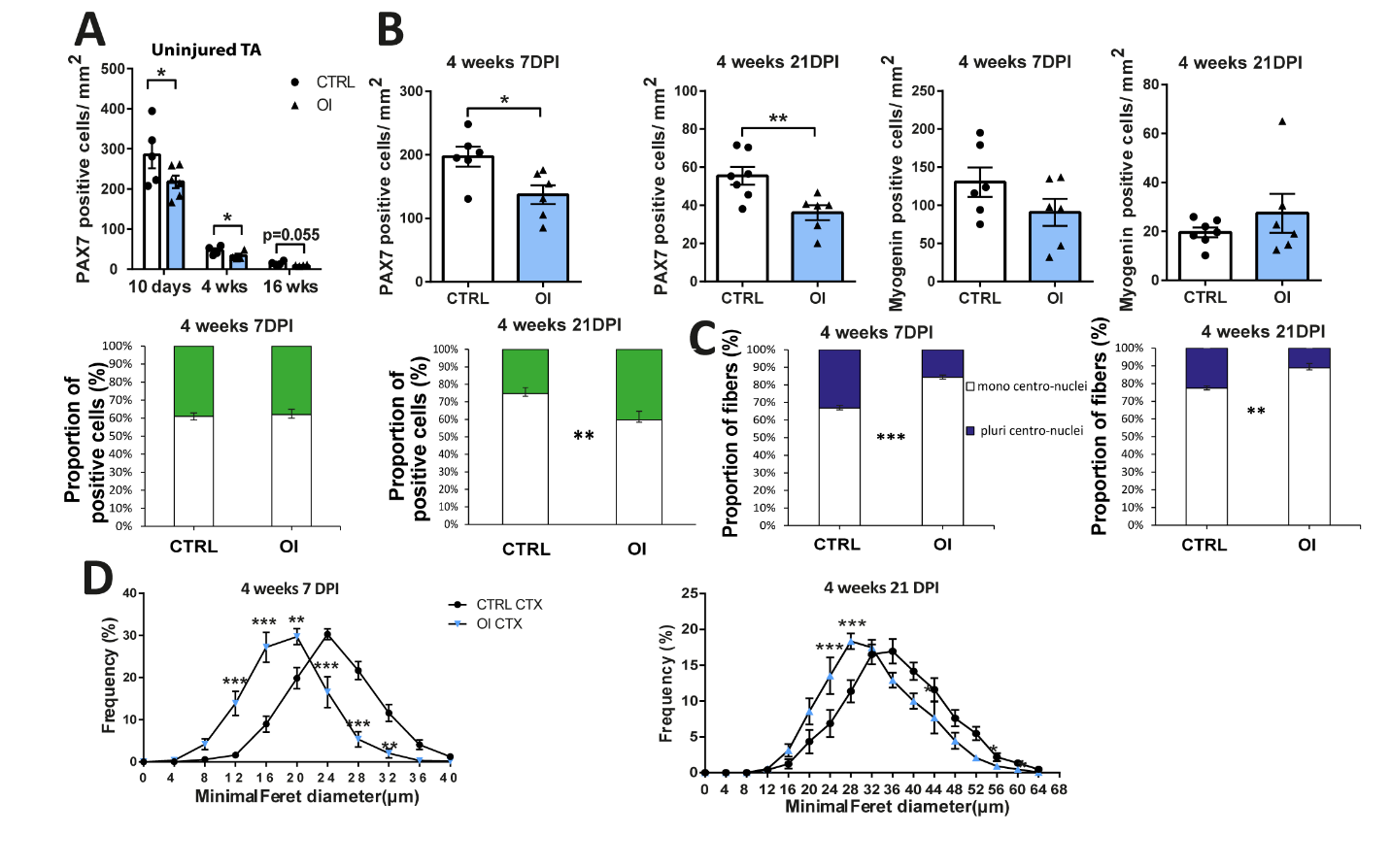
^

**Supplemental Figure 1: Impact of transient neonatal exposure to high oxygen on muscle stem cell pool and regenerative capacity at 4 weeks in female rats**. (A) Density of PAX7 positive cells per mm^2^ in the tibialis anterior (TA) from room air control (CTRL) and neonatal oxygen-induced injury (OI) female rats at 10 days, 4- and 16 weeks (wks). Animals from the neonatal oxygen-induced injury (OI) or room air control (CTRL) groups were injured, and muscle regeneration was assessed at 7- and 21 days post-injury (DPI). (B) Density of muscle stem cells (PAX7 positive) and differentiated myoblasts (Myogenin positive) per mm^2^, and the proportion of PAX7/Myogenin cells in the TA from 4 wks old female rats at 7- and 21 days post-injury (DPI). (C) Proportion of pluri- and mono-centronucleated fibers in the TA from 4 wks CTRL and OI female rats at 7- and 21 DPI. (D) Minimal Feret’s diameter of centronucleated myofibers of the TA from 4 wks old CTRL and OI female rats at 7- and 21 DPI. Error bars represent means ± SEM; (A) n=4-6, and (B-K) n=6-7 per group. (A-C) Statistical analyses were performed using student t-test to compare OI vs CTRL or (D) two-way ANOVA—testing the effects of condition (OI vs. CTRL) and different diameter size—followed by Sidak post-hoc test. *p<0.05; **p<0.01; *** p<0.001 vs. group indicated.


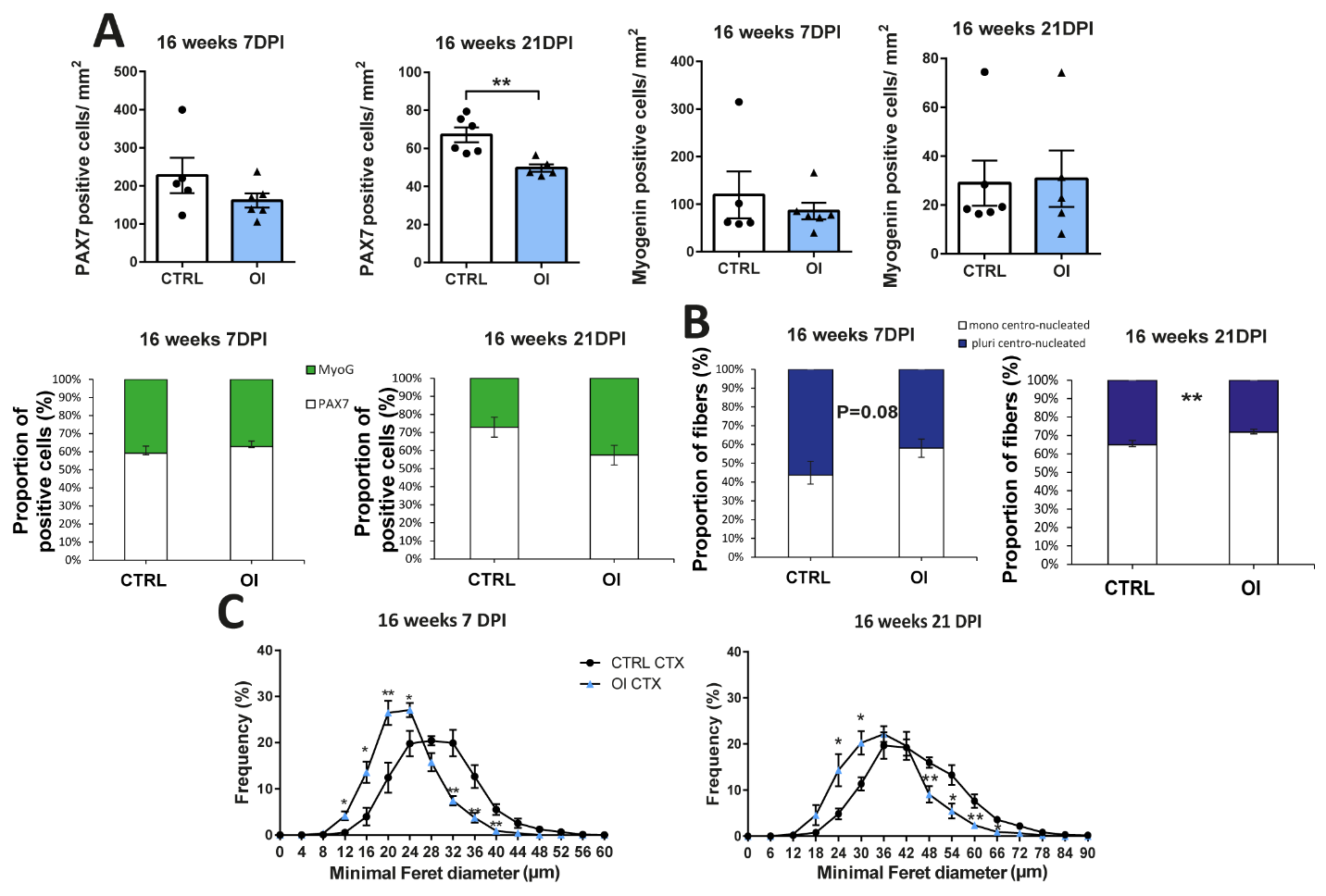


**Supplemental Figure 2: Impact of transient neonatal exposure to high oxygen on muscle regeneration capacity in 16-weeks-old male rats**. (A) Number of muscle stem cells (PAX7-positive) differentiated myoblasts (Myogenin-positive) per mm^2^, and proportion of PAX7 and Myogenin positive cells in the tibialis anterior (TA) of 16 weeks (wks) old male rats at 7- and 21 days post-injury (DPI). (B) Proportion of pluri- and mono-centronucleated fibers in the TA of 16 wks CTRL and OI male rats at 7- and 21 DPI. (C) Minimal Feret’s diameter of centronucleated myofibers in the TA of 16 wks CTRL and OI male rats at 7- and 21 DPI. Error bars represent means ± SEM; n=5-6 per group. (A-B) Statistical analyses were performed using student t-test to compare OI vs CTRL or (C) two-way ANOVA—testing the effects of condition (OI vs. CTRL) and different diameter size—followed by Sidak post-hoc test. *p<0.05; **p<0.01 vs. group indicated.


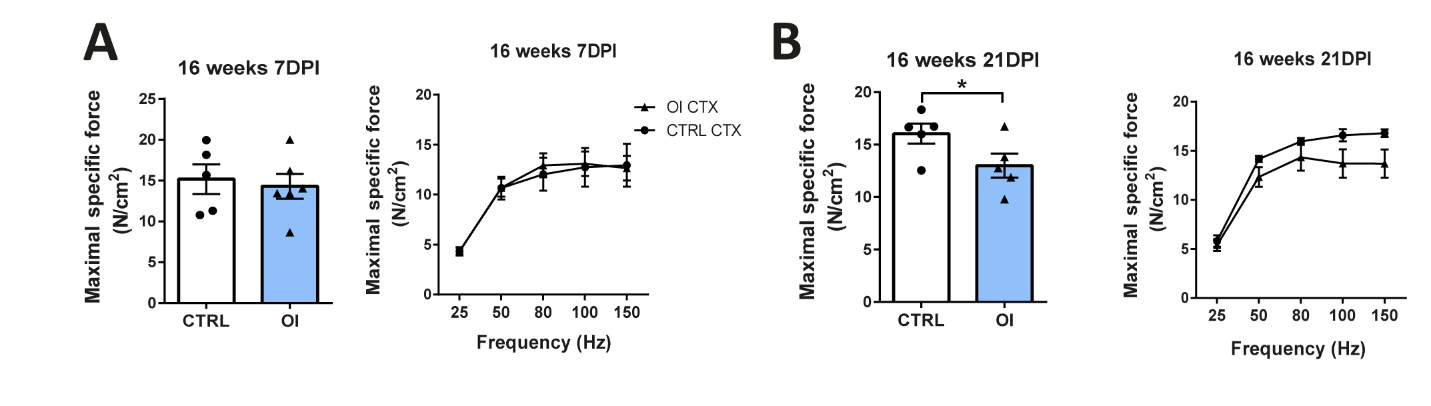


**Supplemental Figure 3: Impact of transient neonatal exposure to high oxygen on muscle contractile properties at 16 weeks in male rats.** (A,B) Maximal specific force, and force-frequency curve of the extensor digitorum longus (EDL) muscle from 16 weeks (wks) old room air control (CTRL) vs. neonatal oxygen-induced injury (OI) male rats at 7- and 21 days post-injury (DPI). Error bars represent means ± SEM; n=5-6 per group. Statistical analyses were performed using student t-test to compare OI vs CTRL groups.

**
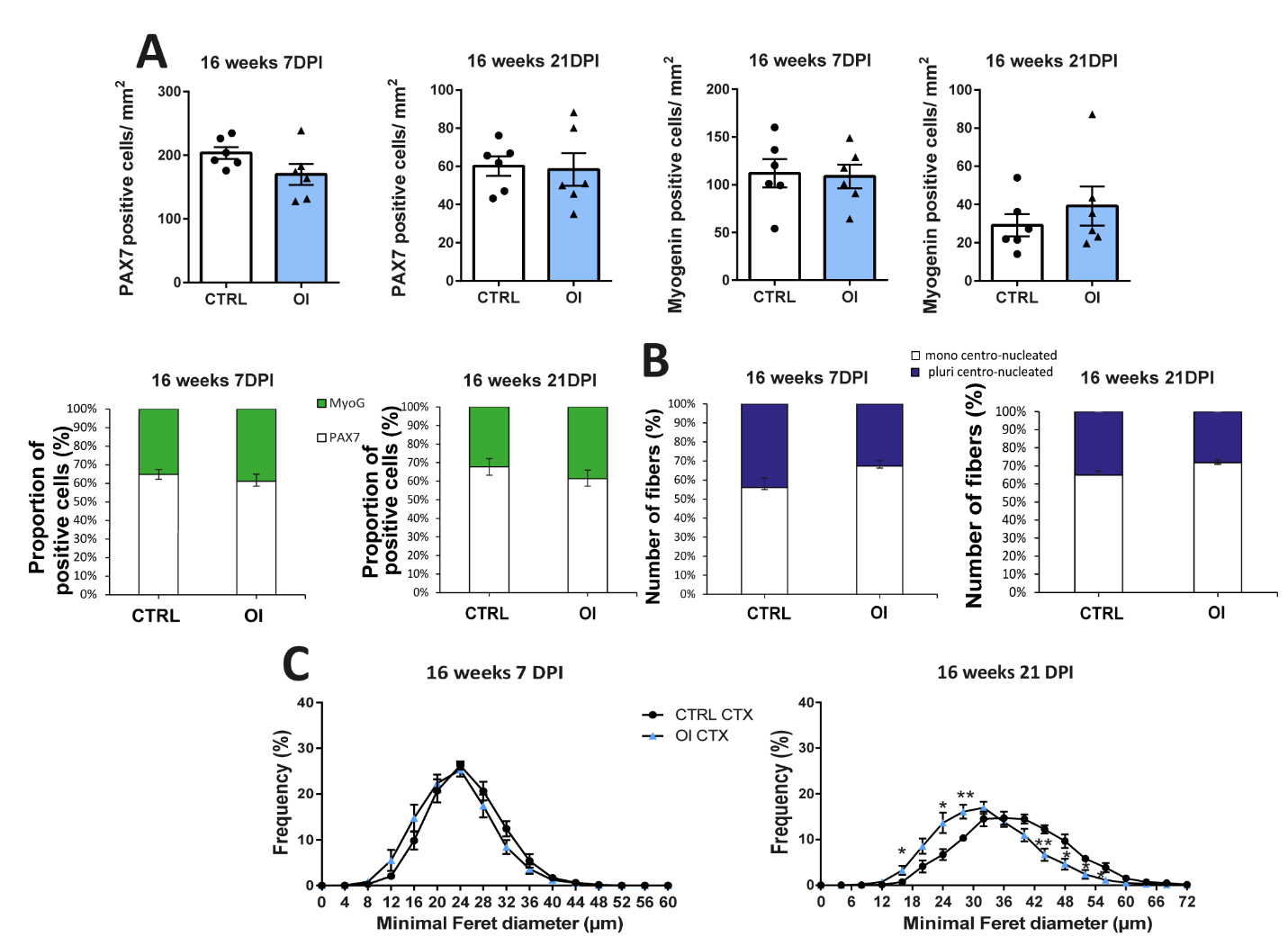
**

**Supplemental Figure 4: Impact of transient neonatal exposure to high oxygen on muscle regeneration capacity in 16 weeks old female rats**. (A) Number of muscle stem cells (PAX7 positive) differentiated myoblasts (Myogenin positive) per mm^2^, as well as the proportion of PAX7/Myogenin cells in the tibialis anterior (TA) of 16 weeks (wks) old room air control (CTRL) and neonatal oxygen-induced injury (OI) female rats at 7- and 21 days post-injury (DPI). (B) Proportion of pluri- and mono-centronucleated fibers in the TA from 16 wks CTRL and OI female rats at 7- and 21 DPI. (C) Minimal Feret’s diameter of centronucleated myofibers of the TA from 16 wks CTRL and OI female rats at 7- and 21 DPI. Error bars represent means ± SEM; n=5-6 per group. (A-B) Statistical analyses were performed using student t-test to compare OI vs CTRL or (C) two-way ANOVA—testing the effects of condition (OI vs. CTRL) and different diameter size—followed by Sidak post-hoc test. *p<0.05; **p<0.01 vs. group indicated.


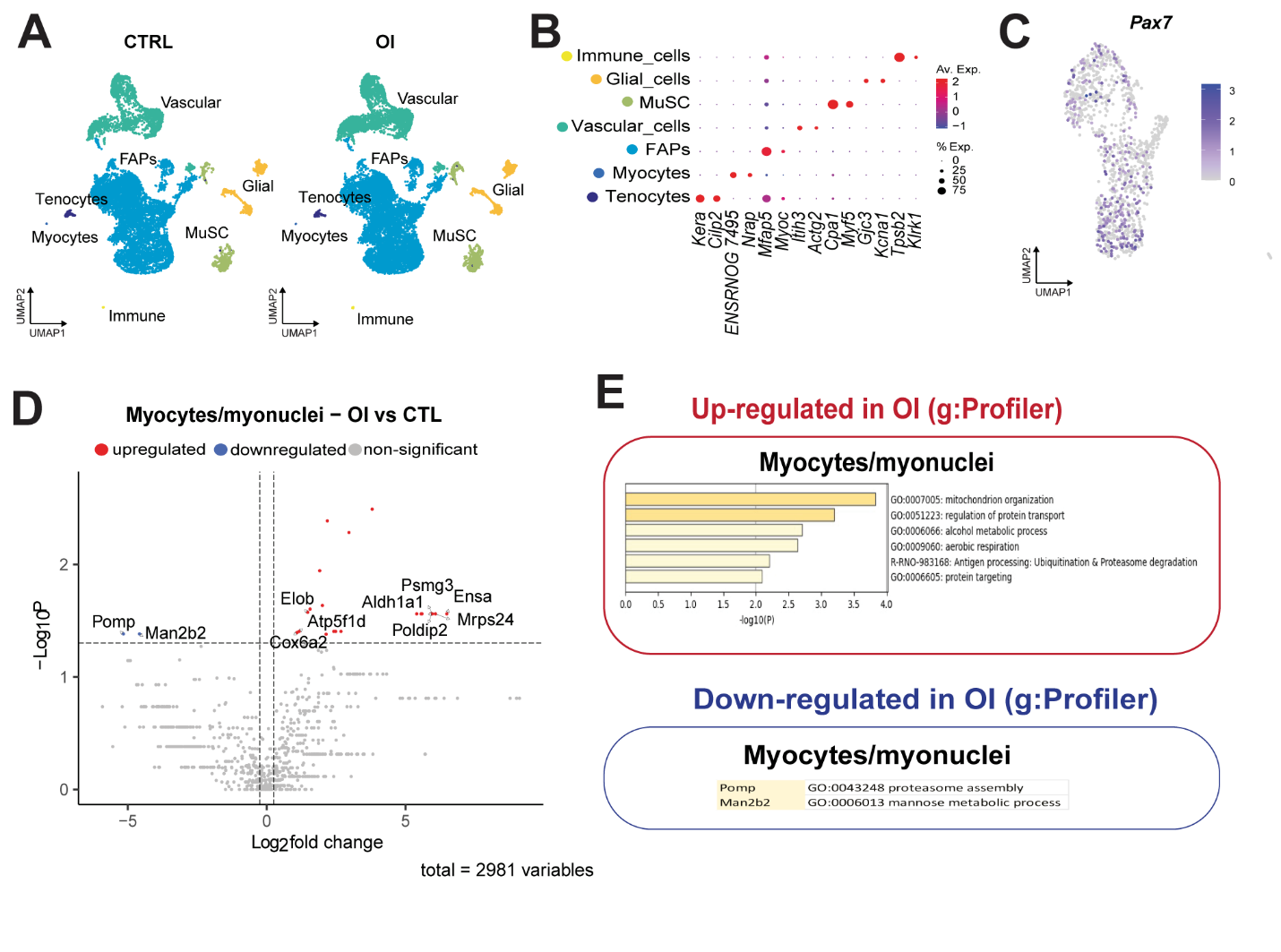


**Supplemental Figure 5: scRNA-seq analysis of muscle stem cells from control and oxygen-induced injury male rats.** (A) UMAP of the room air control (CTRL) and neonatal oxygen-induced injury (OI) male rats cell populations at 4 weeks. (B) Dot plot diagram showing markers genes expressed in each cluster of cells population. (C) UMAP embeddings CTRL and OI male rat cells displaying gene expression of *PAX7*. (D) Volcano plot depicting differentially expressed genes (DEGs) downregulated or upregulated in OI cells vs CTRL myocytes/myonuclei subset. (E) Functional enrichment analysis with GO Biological Processes analysis using DEGs downregulated and upregulated in OI vs CTRL cells. The X-axis represents the –log10(P-value). N=2 independent biological samples/group.

**
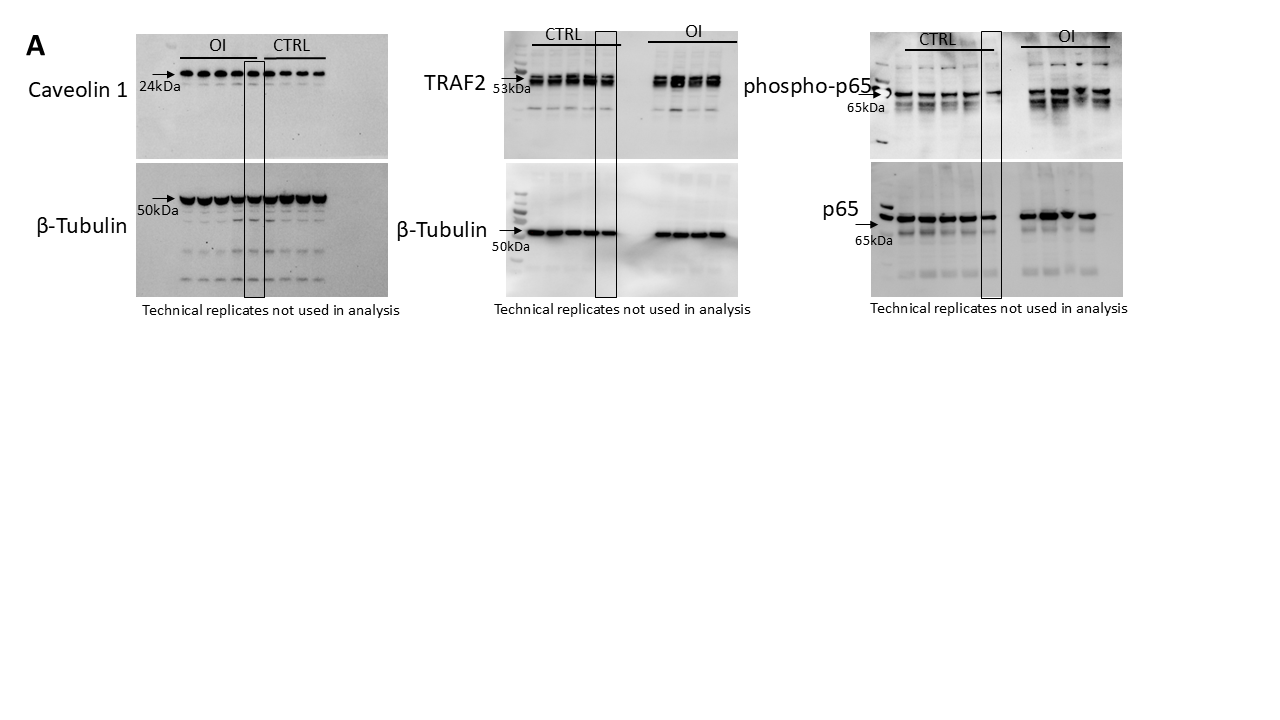
**

**
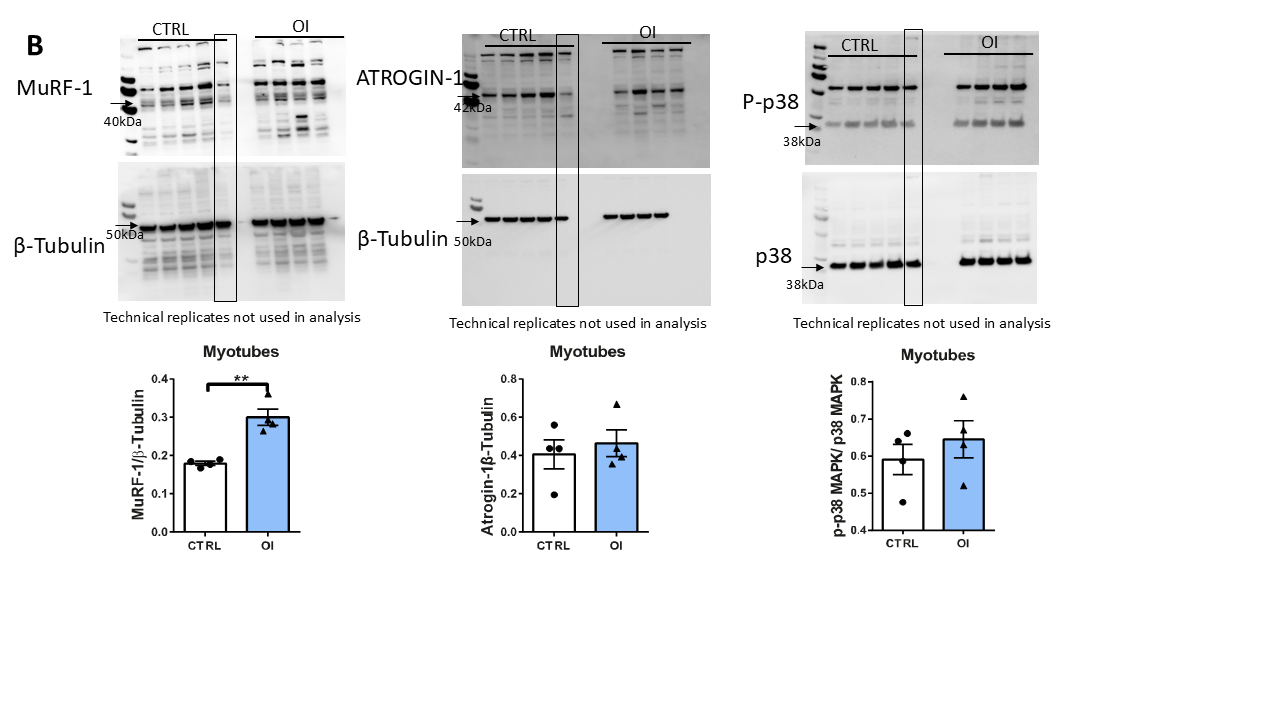

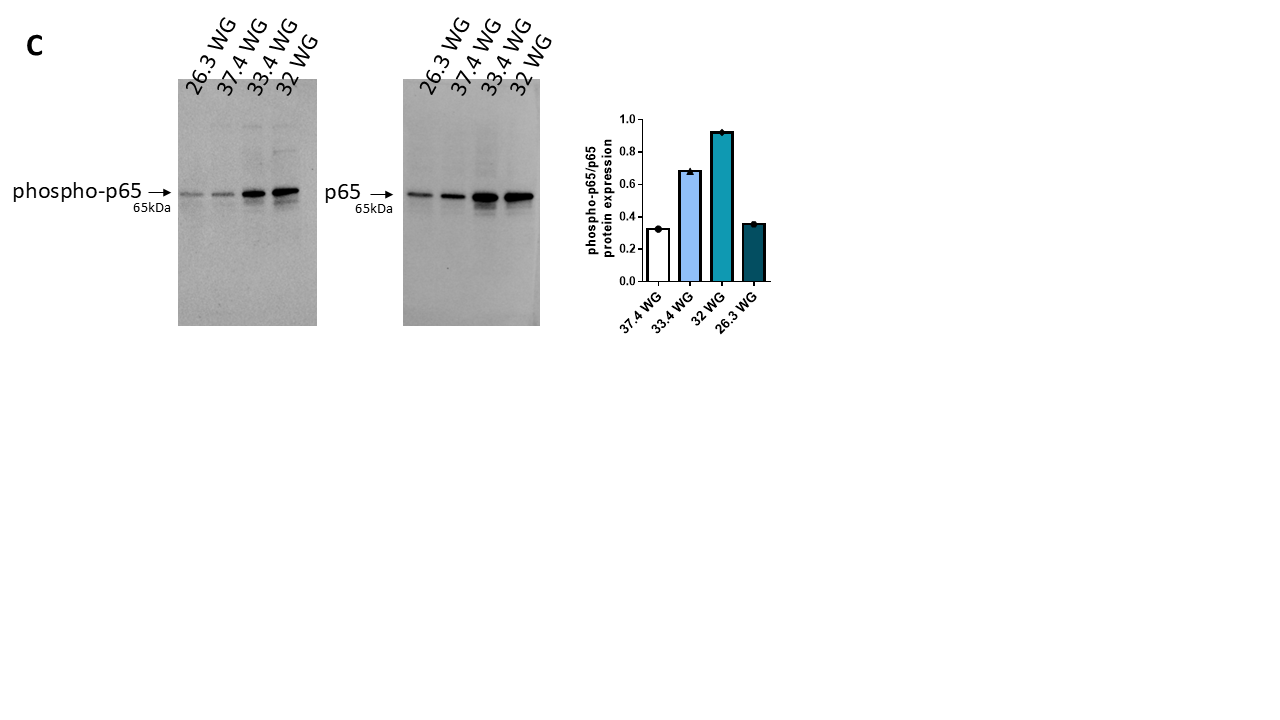
**

**Supplemental Figure 6: Quantification by western blot of Caveolin 1, TRAF2, atrogenes and NF-kB protein.** (A) Images of the full unedited western blots showing the protein expression of Caveolin1, TRAF2, phospho-p65, p65 and β-Tubulin (as loading control) from in vitro myotubes of 4 weeks old CTRL and OI male rats. (B) Images of the full unedited Western blots and quantification of the protein expression, Muscle RING Finger 1(MuRF-1), Atrogin-1, phospho-p38 MAPK, p38 MAPK and β-Tubulin (as loading control) from in vitro myotubes of 4 weeks old CTRL and OI male rats. (C) Images of the full unedited Western blots and quantification of the protein expression of phospho-p65, p65 from in vitro myotubes isolated from deceased babies born preterm (26.3, 32 and 33.4 weeks of gestation (WG) and at term (37.4 WG).


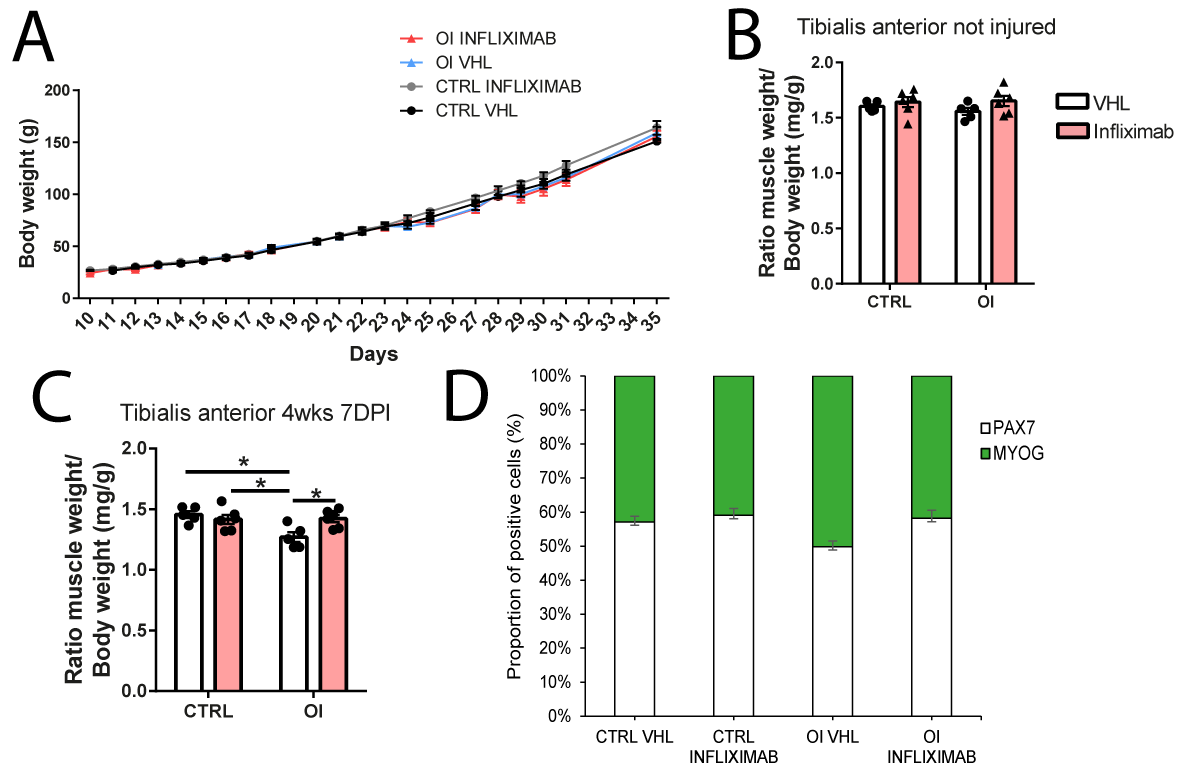


**Supplemental Figure 7:** **TNF-α inhibitor impact on the body weight and muscle weight of juvenile male rats.** (A) Body weight (g) of room air control (CTRL) vs. neonatal oxygen-induced injury (OI) male rats treated with infliximab or vehicle (VHL). Ratio of muscle weight (mg) on body weight (g) from (B) uninjured tibialis anterior (TA) at 4 weeks (wks) and (C) TA after 7 days post-injury (DPI) (D) proportion of PAX7 and Myogenin positive cells of the TA from CTRL vs OI male rats at 4 wks of age and 7 DPI treated with infliximab or VHL. Error bars represent means ± SEM; n=6 independent biological sample per group for CTRL and OI infliximab; n=5 for CTRL and OI VHL. Statistical analyses were performed using Two-way ANOVA —testing the effects of condition (OI vs. CTRL) and treatment (VEH vs. Infliximab) and their interaction—followed by Tukey’s post-hoc test. *p<0.05 vs. group indicated.
