# Supplementary material for: Preterm Birth Conditions Alter Muscle Stem Cells and Their Niche, Causing Lasting Impairments in Muscle Regeneration and Function
Source: J Cachexia Sarcopenia Muscle. 2025 Sep 16;16(5):e70058. doi: 10.1002/jcsm.70058 (PMC12439181; doi:10.1002/jcsm.70058)
Supplement: Supplementary file 2 — Data S1: Supplementary Information [file JCSM-16-e70058-s002.docx]

**Supporting Information for**

**Preterm birth conditions alter muscle stem cells and their niche, causing lasting impairments in muscle regeneration and function.**

Alyson Deprez^1,2^, Thomas Molina^1,2^, Gael Cagnone^1^, Pauline Garcia^1^, Séverine Leclerc^1^, Anik Cloutier^1^, Rebecca Desaulniers^1,2^, Benjamin Ellezam^1^, Anne Monique Nuyt^1,3*^, Nicolas A. Dumont^1,4*^

**Supplemental materials and methods**

Myofibers isolation

Animals at 4 weeks of age were anesthetized with i.p. injection of pentobarbital sodium (50 mg/kg) and maintained anesthetized during the dissection of the flexor digitorum brevis (FDB) from both paws. FDB were washed in NaCl 0.9% solution to remove blood and they were digested in collagenase I solution (DMEM with 3.5mg/ml collagenase I and 1% of penicillin streptomycin) at 37°C for 50 minutes. Then, FDB were transferred to a 6-well plate and placed under an inverted microscope and myofibers were isolated by gently triturate FDB muscle. After 1 hour of washing, myofibers were put in 24 wells plate, no more than 3 myofibers per well, in culture medium (DMEM + 20% fetal bovine serum (FBS) + 1% Chicken Embryo Extract + 1% Fibroblast Growth Factor-2 + 1% penicillin streptomycin). Myofibers were cultured for 0h, 40h, or 60h.

Immunostaining

Muscles were embedded in Tissue-Tek® optimal cutting temperature compound (OCT compound, Sakura Finetek USA Inc., Torrance, CA) and frozen in 2-Methylbutane (Sigma-Aldrich, Missouri, USA) cooled in liquid nitrogen. Immunostainings were performed on transversal cryosections (10 μm) from proximal, medial, and distal parts of the TA or on EDL single myofibers muscles. Samples were incubated with the following primary antibodies overnight at 4°C mouse anti-Pax7 (Developmental Studies Hybridoma Bank (DSHB), created by the NICHD of the NIH and maintained at The University of Iowa, Department of Biology, Iowa City, IA 52242; dilution 1:3), rabbit anti-MyoD (Santa cruz, SC-304, 1:500), rabbit anti-myogenin (Abcam, Toronto, Canada, a124800, 1:500), Rabbit anti-laminin (Abcam, Toronto, Canada, ab11575, 1:1,000), mouse anti-dystrophin (DSHB, Iowa, United States, manex1011b, 1:3), mouse anti-embryonic myosin heavy chain (F1.652, DSHB, Iowa, United States, 1:3), Rat anti-KI67 (Invitrogen, 14-5698-82, clone SolA15, 1:500), mouse anti-Myosin Heavy Chain (DSHB, Iowa, United States, MF-20, 1:10). Samples were washed and incubated with secondary fluorescent antibodies (1:1,000) for 1 hour at room temperature. Slides were mounted using Dako fluorescence mounting medium (Dako). Immunofluorescence pictures of samples were taken with EVOS M5000 (Thermo Fisher Scientific, EVOS M5000 Software Revision 1.5.1500.493) or Leica DMi 8 microscope or Leica TCS SP8 DLS confocal microscope. Pictures were analysed using ImageJ software (NIH, Bethestda, MD). For the Pax7 positive cells, myogenin density evaluation, number of centronucleated fibers and the minimal feret’s diameter (minimal distance between the two parallel tangents of the muscle fiber; as described previously^10^ a minimum of 6 images were randomly selected from different parts of the muscle section in injured area (10X magnification; Leica DMi8 microscope). For the minimal feret’s diameter, a minimum of 200 fibers per muscle per rat were quantified. For myotube analysis, each condition 10 images (20X magnification) were examined with the Myotubes Analyzer working on the open-source Matlab (MATLAB R2021a, MathWorks) app^S9^. All analysis were performed by a single experimenter blinded to the identity of the samples.

Western Blot

Western blot procedure was adapted from a protocol described previously^10^. Membranes were blocked (5% BSA) and incubated with the mouse anti-Caveolin-1 (BD transduction laboratories, 610406, 1:1,000), rabbit anti-TRAF2 (Cell signaling, 90861 T, 1/1,000), rabbit anti-MuRF1 (ProteinTech, 55456-1-AP, 1/1,000), rabbit anti-Atrogin-1 (Abcam, ab168372, 1/1,000), mouse anti-p65(Cell signaling, 6956S, 1/1,000), rabbit anti-phospho-p65 (Cell signaling, 3031S, 1/1,000)and the mouse anti-β-tubulin (Sigma, T0198). Corresponding secondary horseradish peroxidase-conjugated antibodies (rabbit anti-mouse or goat anti-rabbit, Abcam) were added (1:2,000) to the membrane for 1 hour at room temperature. Immunoreactivity was detected using the ECL Western blot detection (Pierce™ ECL Plus Western Blotting Substrate, Thermo Fisher, 32132) in chemidoc (Bio-RAD). Protein expression was normalized to β-tubulin.

Elisa

Quantification of TNF-α protein levels in the tibialis anterior from male rat at 4 weeks was performed using ELISA TNF-α Kit (Thermo Fisher Scientific, ERA57RB) according to manufacturer instruction.

Ex vivo muscle functional testing

For *ex vivo* assessment of muscle function, animals were weighted and anesthetized with i.p. injection of pentobarbital sodium (50 mg/kg) and maintained anesthetized during the dissection to keep the blood perfusion of the EDL. The proximal and distal tendons of the EDL muscles were attached with a 3-0 silk suture. Muscles were carefully dissected and placed in the 300A Test System organ bath (Aurora Scientific Inc., Ontario, Canada) filled with buffered physiological salt solution (Krebs-Ringer supplemented with glucose) continuously perfused with carbogen bubbling (5% CO_2_, 95% O_2_), and thermostatically maintained at 25°C to obtain optimal muscle performance (lower fatigue) ^S10^. Optimal muscle length was determined by stimulating muscle at 1 Hz and adjusting the length until the maximal twitch tension is reached. Thereafter, muscles were stimulated (25 V, 500 ms) at increasing frequencies: 25, 50, 80, 100, 150 Hz. The muscle was allowed to rest for 3 min between two stimulations^S11^. The specific muscle force was calculated as follow: [maximal force × optimal fiber length (0.44 x muscle length) × muscle density (1.06 g/cm^3^)]/muscle mass^S12^. For muscle fatigability, the EDL was subjected to 50 Hz stimulation every 2 seconds over 3 min^S11^. At the end of the protocol, the length and weight of the muscle were measured. Data were analyzed with Dynamic Muscle Data Analysis software version 6.1 (Aurora Scientific, Inc.).

Bioinformatics analysis

A total of 13,117 cells were obtained after UMI deconvolution using Cell Ranger Multi V (10x Genomics). The sample origin of the cells that remained undetermined after CMO demultiplexing (i.e. negative) was recovered using the VireoSNP package (vireoSNP-0.5.7). Following Vireo SNP donor classification, 10,469 cells were assigned to the CTRL group (Rep 1 = 3,149 cells, Rep 2 = 7,320 cells) and 13,283 cells to the OI group (Rep 1 = 6,221 cells, Rep 2 = 7,062 cells).

After quality control and cell filtering based on mitochondrial and total gene expression distribution (<30%), UMAP dimensionality reduction and clustering were performed on SCT transformed counts using Seurat V5^S13^. Main cell type annotation was done based on the differential expression of known marker genes as well as the prediction from SingleR classification algorithm^S14^. The annotated muscle and MuSC clusters were then integrated to the Myogenesis cell Atlas of McKellar et al, 2021^15^ based on SCTransformed counts using the Seurat Anchor based integration function. Single-cell gene expression was visualized using the DotPlot and FeaturePlot functions from Seurat V5. Trajectory analysis was performed using Monocle3 and imbalance score was computed using the condiment package^S15^. Pathway enrichment analysis on differentially expressed genes was performed using gProfiler^S16^ and enrichR^S17^. Pathway activity scores were computed on SCT normalized counts with GSVA (Gene Set Variation Analysis) ^S18^ and represented as log2 FC (from Seurat FindMarker) using the Heatmap.2 function from gplots R package (gplots package - RDocumentation). To identify perturbed cell-cell communication pathways related to inflammation (immune cells), differential connectomics analysis between CTRL and OI cells was performed on differentially expressed ligand-receptor pairs (based on the FANTOM5 database) using the Connectome package^S19^.

**Supplemental References**

S1. Novak JS, et al. Human muscle stem cells are refractory to aging. Aging Cell 20, e13411 (2021).

S2 Garcia SM, et al. High-Yield Purification, Preservation, and Serial Transplantation of Human Satellite Cells. Stem Cell Reports 10, 1160-1174 (2018).

S3 Latil M, et al. Skeletal muscle stem cells adopt a dormant cell state post mortem and retain regenerative capacity. Nat Commun 3, 903 (2012).

S4 Sasarman F, Karpati G, Shoubridge EA. Nuclear genetic control of mitochondrial translation in skeletal muscle revealed in patients with mitochondrial myopathy. Hum Mol Genet 11, 1669-1681 (2002).

S5 Conte TC, et al. Clearance of defective muscle stem cells by senolytics restores myogenesis in myotonic dystrophy type 1. Nat Commun 14, 4033 (2023).

S6 Karson A, Demirtaş T, Bayramgürler D, Balci F, Utkan T. Chronic administration of infliximab (TNF-α inhibitor) decreases depression and anxiety-like behaviour in rat model of chronic mild stress. Basic Clin Pharmacol Toxicol 112, 335-340 (2013).

S7 Dumont NA, et al. Dystrophin expression in muscle stem cells regulates their polarity and asymmetric division. Nat Med 21, 1455-1463 (2015).

S8. Taglietti V, et al. Thyroid-stimulating hormone receptor signaling restores skeletal muscle stem cell regeneration in rats with muscular dystrophy. Sci Transl Med 15, eadd5275 (2023).

S9. Noë S, et al. The Myotube Analyzer: how to assess myogenic features in muscle stem cells. Skelet Muscle 12, 12 (2022).

S10 Segal SS, Faulkner JA, White TP. Skeletal muscle fatigue in vitro is temperature dependent. J Appl Physiol (1985) 61, 660-665 (1986).

S11. Andrich DE, et al. Altered Lipid Metabolism Impairs Skeletal Muscle Force in Young Rats Submitted to a Short-Term High-Fat Diet. Front Physiol 9, 1327 (2018).

S12 Brooks SV, Faulkner JA. Contractile properties of skeletal muscles from young, adult and aged mice. J Physiol 404, 71-82 (1988).

S13. Hao Y, et al. Dictionary learning for integrative, multimodal and scalable single-cell analysis. Nat Biotechnol 42, 293-304 (2024).

S14. Aran D, et al. Reference-based analysis of lung single-cell sequencing reveals a transitional profibrotic macrophage. Nat Immunol 20, 163-172 (2019).

S15. Roux de Bézieux H, Van den Berge K, Street K, Dudoit S. Trajectory inference across multiple conditions with condiments. Nat Commun 15, 833 (2024).

S16 Reimand J, et al. g:Profiler-a web server for functional interpretation of gene lists (2016 update). Nucleic Acids Res 44, W83-89 (2016).

S17. Xie Z, et al. Gene Set Knowledge Discovery with Enrichr. Curr Protoc 1, e90 (2021).

S18. Hänzelmann S, Castelo R, Guinney J. GSVA: gene set variation analysis for microarray and RNA-seq data. BMC Bioinformatics 14, 7 (2013).

S19. Raredon MSB, et al. Computation and visualization of cell-cell signaling topologies in single-cell systems data using Connectome. Sci Rep 12, 4187 (2022).

S20 Tetri LH, et al. Sex-Specific Skeletal Muscle Fatigability and Decreased Mitochondrial Oxidative Capacity in Adult Rats Exposed to Postnatal Hyperoxia. Front Physiol 9, 326 (2018).

S21. O'Reilly M, Thébaud B. Animal models of bronchopulmonary dysplasia. The term rat models. Am J Physiol Lung Cell Mol Physiol 307, L948-958 (2014).

S22. Ravizzoni Dartora D, et al. Cardiac Left Ventricle Mitochondrial Dysfunction After Neonatal Exposure to Hyperoxia: Relevance for Cardiomyopathy After Preterm Birth. Hypertension 79, 575-587 (2022).

S23. Yoshimoto Y, Ikemoto-Uezumi M, Hitachi K, Fukada SI, Uezumi A. Methods for Accurate Assessment of Myofiber Maturity During Skeletal Muscle Regeneration. Front Cell Dev Biol 8, 267 (2020).

S24. Ganassi M, Zammit PS. Involvement of muscle satellite cell dysfunction in neuromuscular disorders: Expanding the portfolio of satellite cell-opathies. Eur J Transl Myol 32, (2022).

S25. Feichtinger RG, et al. Biallelic variants in the transcription factor PAX7 are a new genetic cause of myopathy. Genet Med 21, 2521-2531 (2019).

S26 Byun MK, Cho EN, Chang J, Ahn CM, Kim HJ. Sarcopenia correlates with systemic inflammation in COPD. Int J Chron Obstruct Pulmon Dis 12, 669-675 (2017).

S27 Wei J, Xiong XF, Lin YH, Zheng BX, Cheng DY. Association between serum interleukin-6 concentrations and chronic obstructive pulmonary disease: a systematic review and meta-analysis. PeerJ 3, e1199 (2015).

S28 Mesquita T, Lin YN, Ibrahim A. Chronic low-grade inflammation in heart failure with preserved ejection fraction. Aging Cell 20, e13453 (2021).

S29. Balnis J, et al. Deaccelerated Myogenesis and Autophagy in Genetically Induced Pulmonary Emphysema. Am J Respir Cell Mol Biol 66, 623-637 (2022).

S30. Wei J, Xiong XF, Lin YH, Zheng BX, Cheng DY. Association between serum interleukin-6 concentrations and chronic obstructive pulmonary disease: a systematic review and meta-analysis. PeerJ 3, e1199 (2015).

S31. Chen Q, et al. CD44(high) alveolar type II cells show stem cell properties during steady-state alveolar homeostasis. Am J Physiol Lung Cell Mol Physiol 313, L41-l51 (2017).

S32 O'Neill KM, et al. NOX4 is a major regulator of cord blood-derived endothelial colony-forming cells which promotes post-ischaemic revascularization. Cardiovasc Res 116, 393-405 (2020).

S33. Bakkar N, et al. IKK/NF-kappaB regulates skeletal myogenesis via a signaling switch to inhibit differentiation and promote mitochondrial biogenesis. J Cell Biol 180, 787-802 (2008).

S34. Guttridge DC, Albanese C, Reuther JY, Pestell RG, Baldwin AS, Jr. NF-kappaB controls cell growth and differentiation through transcriptional regulation of cyclin D1. Mol Cell Biol 19, 5785-5799 (1999).

S35 Capozza F, et al. Muscle-specific interaction of caveolin isoforms: differential complex formation between caveolins in fibroblastic vs. muscle cells. Am J Physiol Cell Physiol 288, C677-691 (2005).

S36. Barresi V, Grosso M, Barresi G. Immunohistochemical evidence of caveolin-1 expression in the human fetal and neonatal striated muscle and absence in the adult's. Appl Immunohistochem Mol Morphol 16, 267-273 (2008).

S37. Baker N, Tuan RS. The less-often-traveled surface of stem cells: caveolin-1 and caveolae in stem cells, tissue repair and regeneration. Stem Cell Res Ther 4, 90 (2013).

S38. Bae GD, Park EY, Kim K, Jang SE, Jun HS, Oh YS. Upregulation of caveolin-1 and its colocalization with cytokine receptors contributes to beta cell apoptosis. Sci Rep 9, 16785 (2019).

S39. Catalán V, et al. Expression of caveolin-1 in human adipose tissue is upregulated in obesity and obesity-associated type 2 diabetes mellitus and related to inflammation. Clin Endocrinol (Oxf) 68, 213-219 (2008).

S40 Brack AS, Bildsoe H, Hughes SM. Evidence that satellite cell decrement contributes to preferential decline in nuclear number from large fibres during murine age-related muscle atrophy. J Cell Sci 118, 4813-4821 (2005).

S41. Whaley KG, et al. Multicenter Cohort Study of Infliximab Pharmacokinetics and Therapy Response in Pediatric Acute Severe Ulcerative Colitis. Clin Gastroenterol Hepatol 21, 1338-1347 (2023).

S42. Conrad MA, Kelsen JR. The Treatment of Pediatric Inflammatory Bowel Disease with Biologic Therapies. Curr Gastroenterol Rep 22, 36 (2020).

S43. DeBoer MD, et al. Increases in IGF-1 After Anti-TNF-α Therapy Are Associated With Bone and Muscle Accrual in Pediatric Crohn Disease. J Clin Endocrinol Metab 103, 936-945 (2018).

S44. Yzydorczyk C, et al. Neonatal oxygen exposure in rats leads to cardiovascular and renal alterations in adulthood. Hypertension 52, 889-895 (2008).
